# On-Orbit Performance of the *Helioseismic and Magnetic Imager* Instrument onboard the *Solar Dynamics Observatory*

**DOI:** 10.1007/s11207-018-1259-8

**Published:** 2018-02-23

**Authors:** J. T. Hoeksema, C. S. Baldner, R. I. Bush, J. Schou, P. H. Scherrer

**Affiliations:** 1 W.W. Hansen Experimental Physics Laboratory, Stanford University, Stanford, CA 9430 USA; 2 Max-Planck-Institut für Sonnensystemforschung, Justus-von-Liebig-Weg 3, 37077 Göttingen, Germany

**Keywords:** Instrumentation and data management, Instrumental effects, Velocity fields, photosphere, Magnetic fields, photosphere

## Abstract

The *Helioseismic and Magnetic Imager* (HMI) instrument is a major component of NASA's *Solar Dynamics Observatory* (SDO) spacecraft. Since commencement of full regular science operations on 1 May 2010, HMI has operated with remarkable continuity, *e.g.* during the more than five years of the SDO prime mission that ended 30 September 2015, HMI collected 98.4% of all possible 45-second velocity maps; minimizing gaps in these full-disk Dopplergrams is crucial for helioseismology. HMI velocity, intensity, and magnetic-field measurements are used in numerous investigations, so understanding the quality of the data is important. This article describes the calibration measurements used to track the performance of the HMI instrument, and it details trends in important instrument parameters during the prime mission. Regular calibration sequences provide information used to improve and update the calibration of HMI data. The set-point temperature of the instrument front window and optical bench is adjusted regularly to maintain instrument focus, and changes in the temperature-control scheme have been made to improve stability in the observable quantities. The exposure time has been changed to compensate for a 20% decrease in instrument throughput. Measurements of the performance of the shutter and tuning mechanisms show that they are aging as expected and continue to perform according to specification. Parameters of the tunable optical-filter elements are regularly adjusted to account for drifts in the central wavelength. Frequent measurements of changing CCD-camera characteristics, such as gain and flat field, are used to calibrate the observations. Infrequent expected events such as eclipses, transits, and spacecraft off-points interrupt regular instrument operations and provide the opportunity to perform additional calibration. Onboard instrument anomalies are rare and seem to occur quite uniformly in time. The instrument continues to perform very well.

## Introduction

The *Solar Dynamics Observatory* (SDO) with the *Helioseismic and Magnetic Imager* (HMI) instrument onboard was launched 11 February 2010 to provide the observations necessary to understand the sources of solar variability and its impact on the terrestrial environment (Pesnell, Thompson, and Chamberlin, 2012; Scherrer *et al.*, 2012). Since 1 May 2010, the HMI has observed the full disk of the Sun almost continuously to measure the velocity, intensity, and magnetic field in the photosphere (Schou *et al.*, 2012a). As of October 2016, nearly 1100 refereed articles have made use of HMI data. This article describes how the instrument has performed.

HMI operates using two $4096 \times 4096$ CCD cameras to take sequences of polarized filtergrams of the photosphere. The full-disk images, tuned to six wavelengths across the Fe i 6173.3433 Å spectral line in each of six polarization states, are downlinked and combined to determine the basic HMI observable quantities: Doppler velocity, line-of-sight (LoS) magnetic field, line width, line depth, continuum intensity, and the Stokes polarization parameters (Couvidat *et al.*, 2016). More advanced products computed from these observables include vector magnetograms (Hoeksema *et al.*, 2014) and subsurface-flow maps (Zhao *et al.*, 2012).

### HMI Filtergram Data Processing and Calibration

SDO data are collected continuously at a ground station in White Sands, New Mexico, and the HMI and *Atmospheric Imaging Assembly* (AIA) housekeeping and science-data telemetry packets are transferred in near real time to the Joint Science Operations Center (JSOC) Science Data Processing facility at Stanford University. The HMI processing pipeline produces several levels of data products from the incoming 55 megabit-per-second data stream.

The raw HMI bit stream is initially converted into Level-0 images (Lev0), and all of the relevant metadata are extracted.

Image-specific calibrations are applied during the creation of the Level-1 filtergrams. One of the main objectives of this article is to describe these calibrations and the on-orbit measurements made to enable them. CCD overscan rows and columns (extra values returned for pixels that are not part of the image) are removed from the images at this stage, the CCD dark current is subtracted, and a flat field is applied. A limb-finder algorithm estimates the Sun-center location and the solar radius of each image. Another software module is applied to detect cosmic-ray hits and identify bad pixels. The resulting polarized filtergram images, with their lists of bad pixels, are termed Level-1 data (Lev1).

Other corrections (for image distortion, wavelength differences, and polarization cross talk) are made later, at the point when filtergrams are combined during the computation of the scientific observables, as described by Couvidat *et al.* (2016). However, the calibration observations that enable these calibrations are described here.

Initial calibrations of HMI were carried out before launch to assess the performance of the wavelength-filter system (Couvidat *et al.*, 2012b), polarization system (Schou *et al.*, 2012b), and imaging optics (Wachter *et al.*, 2012). Here we detail how the instrument has been operated, monitored, calibrated, and adjusted since launch. Schou *et al.* (2012a), Couvidat *et al.* (2012a), and Couvidat *et al.* (2016) describe the HMI data processing required to compute the observable quantities from the filtergrams. Additional systematic calibration issues determined after launch are addressed by Liu *et al.* (2012) (LoS magnetic field), Hoeksema *et al.* (2014) and Bobra *et al.* (2014) (vector magnetic field), and Kuhn *et al.* (2012) (limb shape).

### Overall HMI Data Recovery

An important requirement for the HMI is high observing continuity, the strongest driver being the need for precise determination of solar-oscillation frequencies for helioseismology.

After two and a half months of commissioning, the HMI instrument formally began full science operations on 1 May 2010, although some data products are available prior to that date. Since then, HMI has operated almost continuously. Most interruptions are either planned, in order to accommodate spacecraft operations and calibrations, or due to unavoidable seasonal eclipses that are a consequence of the SDO geosynchronous orbit.

HMI acquired more than 112 million images from 1 May 2010 to 31 December 2016. Table 1 reports the total number of Level-0 images, as well as the numbers of $4~\mbox{k}\times 4~\mbox{k}$ images that are missing or partially recovered. Images deliberately not collected during the dark phase of eclipses are not reported as missing in the table. About 1.19% of the images were taken with the image stabilization system (ISS) turned off during some spacecraft maneuvers and around the time of eclipses.

**Table 1:** HMI Level-0 image recovery completeness; 1 May 2010 – 31 December 2016.

Parameter	Number of images	Percentage
Total HMI exposures	112,043,265	
Missing images	61,563	0.055%
Partial images	23,698	0.021%

A more relevant statistic may be the number of Dopplergrams recovered during the mission. Dopplergrams, one of the prime HMI observables, are computed every 45 seconds using filtergrams obtained by one of the two HMI cameras. This camera is variously referred to as the front camera, the Doppler camera, or Camera 2. The other camera is called the side camera, vector camera, or Camera 1. As shown in Table 2, more than 98% of all possible Dopplergrams have been recovered during the first five years of the mission. An overall assessment of the quality of each Dopplergram appears in the QUALITY keyword. A zero value for QUALITY indicates that there are no known issues with the data; these are reported as *good* in Table 2. In fact, all HMI data products at every processing level include a QUALITY assessment. Each bit in the QUALITY keyword indicates an issue that might affect the data. The top bit indicates the data are missing, and other non-zero bits indicate lesser quality or explain why data are not present. This is discussed further in Section 5.5.1 and detailed in Appendices E, G, and H. Because sensitivity to various subtle differences in the data collection and processing varies depending on the analysis, the instrument conditions, data-processing details, calibration-procedure versions, and a host of other quantities are all available in keywords.

**Table 2:** Recovery of 45-second HMI Dopplergrams; 1 May 2010 to 31 December 2016.

Parameter	Value	Fraction
Possible 45 s time slots	4,679,040	100.0%
Good Dopplergrams	4,505,062	96.28%
Lower-quality Dopplergrams	95,484	2.04%
Missing Dopplergrams	78,494	1.68%

Table 7 in Appendix A provides details of the Dopplergram recovery rate for each of the first 37 72-day intervals. The lowest percentages ordinarily occur during eclipse seasons in Spring and Fall. The lowest was 96.45% in June – August 2016. The highest was 99.87% in November 2016 – January 2017.

**Figure 1:**
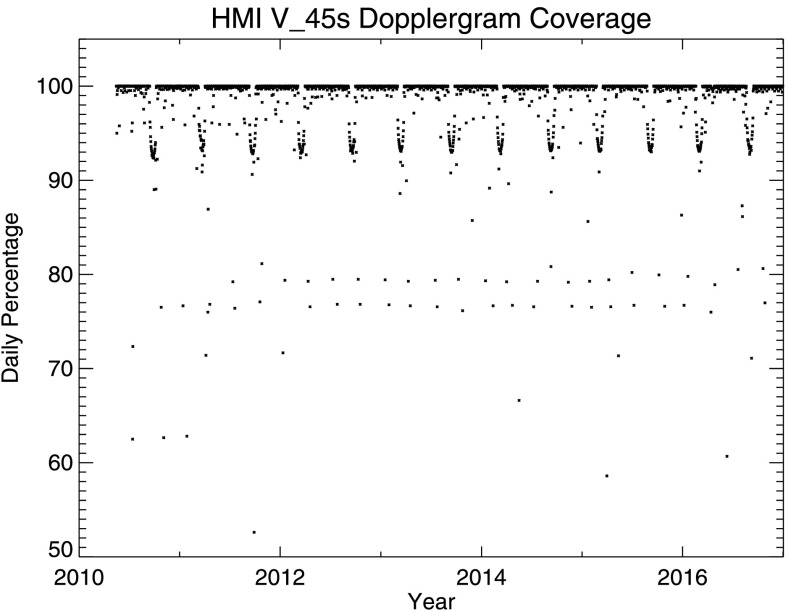
HMI Dopplergram recovery during the mission. The daily percentage of all possible good-quality 45-second Dopplergrams recovered is plotted as a function of time from 1 May 2010 to 31 December 2016. On only 79 days were fewer than 90% of all possible Dopplergrams recovered, and only five days had less than 50% coverage.

Figure 1 shows the percentage of the 1920 possible 45-second Dopplergrams recovered each day. Most days are nearly perfect; only 321 had less than 95% recovery. The semi-annual eclipse seasons can be seen as U-shaped dips to below 95% that extend over several weeks each Spring and Fall when the Earth comes between the spacecraft and the Sun for up to 72 minutes each day. Gaps that can last as long as several hours occur regularly on a few days each quarter when spacecraft operations are scheduled. Occasional dips are deeper when there are special calibrations. On a few occasions, there have been instrument or spacecraft anomalies that have taken longer to recover from. Section 6 provides more information about such events.

### Outline

The purpose of this article is to explain the observations used to calibrate the HMI filtergrams and to characterize the basic performance of the HMI instrument after launch and how it changes with time. This includes consideration of quantities such as throughput, focus, wavelength, and overall data capture, as well as trends in important instrument parameters, such as camera operation, shutter and tuning-motor performance, and subsystem temperatures.

Section 2 describes the routine calibration observations made in order to monitor and optimize the operation of the instrument. Section 3 explains various measurements that show how the instrument has changed over time or responded to events. Section 4 addresses the calibration of the optics and filter systems. In Section 5 we describe the Level-1 processing that produces calibrated filtergrams from Level-0 images, principally the calibrations related to the CCD cameras (flat fields and bad pixels), but also single-pixel corrections for transient problems, such as those caused by cosmic rays. This section also summarizes how characteristics of the image and information about the processing are documented in keywords and encoded in the bits of the QUALITY and CALVER keywords. The implications of events (such as the semiannual eclipses) and occasional onboard anomalies are covered in Section 6. Section 7 gives a summary and discussion of HMI performance. The appendices provide an additional level of detail about observing sequences used for both primary observing and for calibrations, as well as annotated descriptions of more of the keywords for Level-0 and Level-1 filtergrams.

## On-Orbit Calibration Observations

A variety of calibration observations are taken on a regular basis to monitor the evolution of the HMI instrument and maintain optimal performance. This section describes the daily, weekly, bi-weekly, and occasional calibration sequences.

The HMI acquires data using a framelist timeline specification (FTS), or framelist. The FTS defines the filter tuning, polarization state, focus, and timing of each filtergram to be executed in a sequence. The FTS ID is stored in the Level-0 and Level-1 keyword HFTSACID. A roster of the most common frame lists appears in a table in Appendix A.2; more complete listings are provided in Appendix C. The FTS IDs for standard calibration sequences are indicated.

Standard HMI observations were initially obtained with a framelist called Mod C that repeated every 135 seconds. Mod L, a 90-second FTS, replaced Mod C on 13 April 2016. The two versions of Mod C have FTS ID 1001 or 1021; the Mod L HFTSACID is 1022. Some calibration framelists changed when the standard sequences changed.

### Twice-Daily Calibration Sequences

Twice a day, starting at 06 UT and 18 UT, the regular observing sequence is interrupted to run a calibration that includes eight non-standard filtergrams. At these times, near local Noon and Midnight in the orbit, the spacecraft is close to zero radial velocity with respect to the Sun (the exact time varies throughout the year). The sequence consists of four (nearly) true continuum images (tuned such that the filter passbands are about 344 mÅ away from the Fe i line center at rest) taken in two different polarizations, two Calmode images (that is, images taken with the instrument completely defocused in calibration mode), and two dark frames. The continuum frames are not used for calibration purposes, but have been used for some scientific investigations. The Calmode images are used to track the evolution of the throughput of the optical system; the dark images are used to create mean dark frames four times a year (see Section 5). The normal LoS observing sequence in Camera 2 is minimally disturbed. During mod-C (135-second cadence) operations, the FTS ID was 2021; under current mod-L operations, the FTS ID is 2042.

### Weekly Focus Sweeps and PZT Offpoints

Additional calibration sequences are run every week, typically on Tuesdays and Wednesdays around 19:00 UT, although they are sometimes rescheduled or canceled due to conflicts with other events.

**Figure 2:**
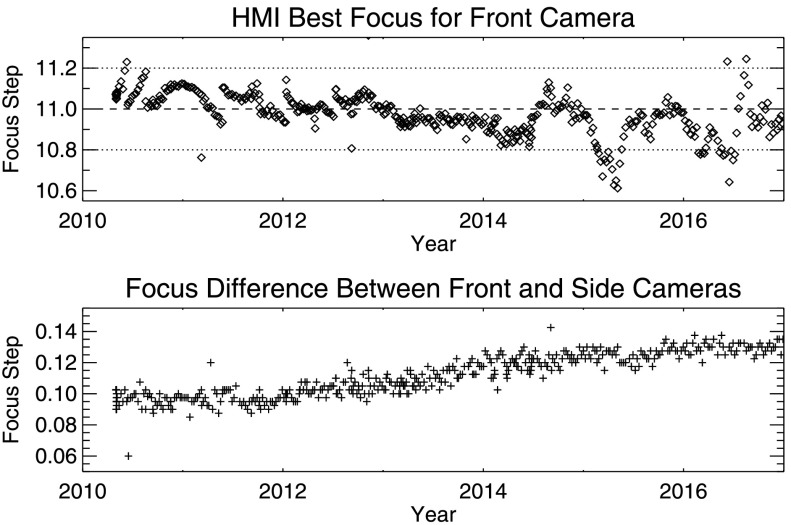
Focus trend observed from the start of the prime mission on 1 May 2010 through the end of 2016 for the HMI front cameras (*top*), and the difference in best focus between the front and side cameras (*bottom*). The temperature of the front window is periodically adjusted to keep the focus near step 11.

Once per week, a focus sweep is taken to determine the instrument's best focus. Two different sequences are used, run on alternate weeks: a full sweep that takes continuum-tuned images at all HMI focus positions (FTS ID 3020, 3040), and a reduced sweep that only uses the seven focus positions around the best-focus position (FTS ID 3023, 3043). The calibration images are processed to determine the focus-block setting that results in the highest image contrast and therefore the optimal focus. Results from these weekly measurements are used to adjust the front-window temperatures to maintain best focus as consistently as possible. The mission-long HMI focus-trend plot for the front camera is presented in the upper panel of Figure 2. The lower panel shows the difference between best-focus position for the front and side cameras. The focus is measured in units of focus steps that are equivalent to 1.04 mm at the CCD camera, about two-thirds of one depth-of-field.

The focus of the two cameras is not identical because of differences in the two light paths. The causes of the relative drift of about 0.03 focus steps over the course of the mission are not fully understood, but might be due to a small (30 micron) change in the relative positions of the CCD detectors that is due to thermal expansion of the optics package.

Another set of calibration images is taken with the Sun deliberately driven off-center using the image stabilization system (ISS). Rather than operating with the normal closed-loop control, the piezo-electric transducers (PZTs) on the guide mirror are driven in a pre-set pattern to move the solar image around on the CCDs. The purpose of these observations is to measure the flat field of each CCD (FTS ID 3021, 3022, 3041, and 3042). This is described further in Section 5.2.

### Bi-weekly Detune Sequence

Every other week, a 60-frame detune sequence is taken to monitor changes in the instrument wavelength-tuning positions and to update the filter-transmission profiles. For the first three months of the regular mission, the detune sequence was run weekly. In this sequence the filter elements are deliberately not co-tuned, *i.e.* they are tuned to a series of 54 different wavelength combinations. The detunes are used to monitor the wavelength drift of the tunable elements. The sequence is taken in calibration mode (Calmode). In Calmode the entrance pupil of the telescope is imaged on the CCDs. The Calmode detunes have been used to determine profiles for the entire duration of the mission. Six dark frames are also collected. The results of these detunes and the periodic adjustments to the best tuning are discussed in Section 4.6. The current FTS ID of this sequence is 3027.

### Occasional Calibrations

Other calibrations are performed on a less regular basis during spacecraft maneuvers that interrupt regular science observations, but provide opportunities to operate the instrument in a unique and useful mode. These include times when SDO is deliberately pointed away from the Sun (offpoints) and times when the spacecraft is rolled from its normal orientation with respect to the solar rotation axis (rolls).

#### Offpoint Flat Fields

Spacecraft offpoint maneuvers are used by all three instruments on SDO for various calibrations. While some procedures are not useful for HMI calibration, quarterly offpoints are used to generate better flat fields. Twenty-two pointings are used, and HMI takes a sequence of continuum-tuned images at a single polarization with a set of varying focus positions. The offpoint flat fields are discussed in more detail in Section 5.2. The current FTS ID for offpoint flat fields is 4031.

#### Roll Calibrations

Roll maneuvers are ordinarily performed twice per year, typically after the eclipse seasons in April and October, when the SDO spacecraft is rotated $360^{\circ }$ around the Sun–spacecraft line. The spacecraft pauses every $22.5^{\circ }$ for approximately twelve minutes. When rolled, the light rays from parts of the solar disk having different rotational velocities take different paths through the instrument filters. This allows us to calibrate the wavelength dependence of the filters (Couvidat *et al.*, 2016). Data taken during these rolls can be also used for (among other things) measuring optical distortion and the shape of the Sun's limb (*e.g.* Kuhn *et al.*, 2012).

Additional roll angles were measured during commissioning in April 2010. A special roll calibration was performed on 23 – 24 March 2016 when SDO was rolled 180^∘^ from its normal orientation for twenty-four hours. During this interval, HMI took detunes every three hours in both normal focus (Obsmode) and completely defocused (Calmode). The FTS IDs for these detunes are 3086 and 3087. The same sets of detunes were taken with the spacecraft in the normal orientation the day before. Analysis verified that the Lyot and Michelson filter-element details (as well as daily temperature variations of the front window) contribute to the 24-hour calibration variations.

#### Other Special Calibrations

SDO has observed two planetary transits since the beginning of the prime mission: one of Venus, and one of Mercury. These transits are useful for calibrating the instrument roll angle, point-spread function, and distortion correction (Couvidat *et al.*, 2016). During each transit, a non-standard observing sequence was run. The LoS observables, taken from the front camera, were produced as normal, but the side camera took continuum-tuned filtergrams in four polarization states for the Venus transit and one polarization for the Mercury transit. The FTS IDs for Venus and Mercury were 4035 and 4039, respectively.

## Trending

It is essential to track the evolution of environmental conditions impacting the HMI observables. This helps with the early detection of problems, characterization of instrument changes and degradation, and the adjustment of the data calibration to maintain the best observables quality possible. Temperatures and voltages are monitored continuously by an autonomous system, and SDO staff are alerted if specified limits are reached. In addition, personnel check the values and trends of various components of the system several times each day to look for odd behavior or to spot problems before they reach cautionary limits. The first two subsections focus primarily on long-term temperature trends measured in the instrument over the course of the mission and on typical daily variations observed during July 2015. The final subsection explains how the plate scale varies in response to temperature changes and how instrument calibration is affected by it.

### Long-Term Instrument Temperature Trends

**Figure 3:**
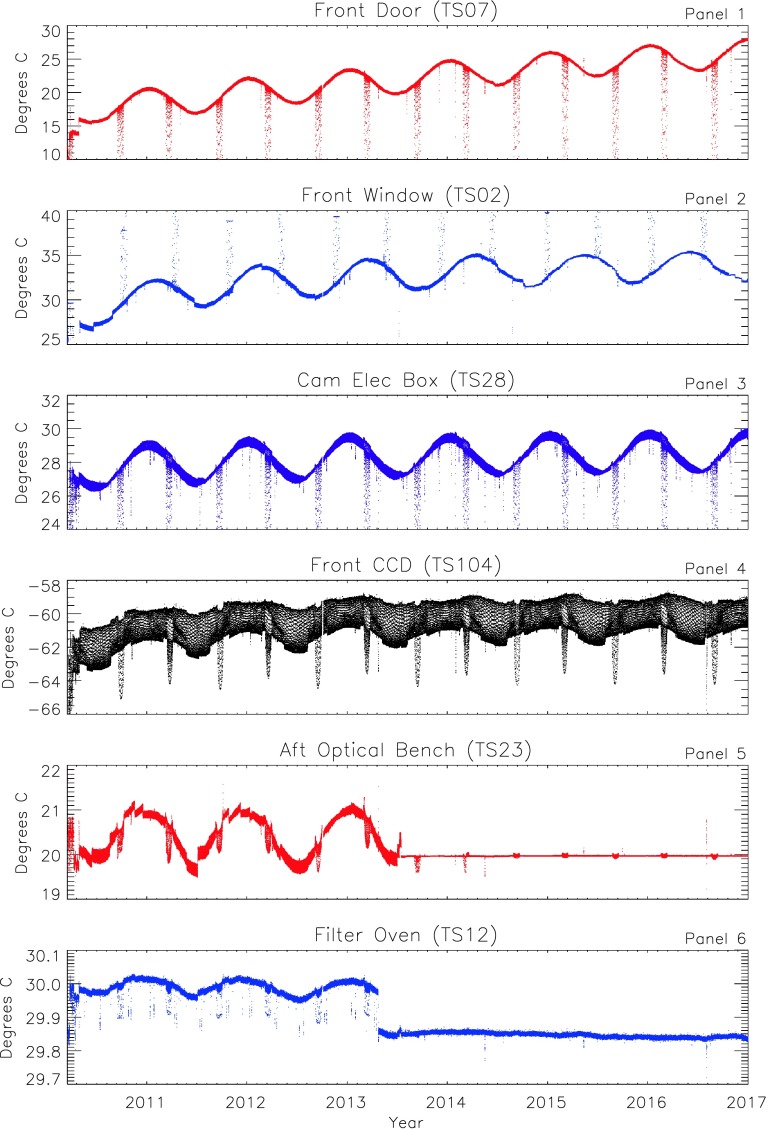
HMI instrument subsystem temperatures from 1 March 2010 through 31 December 2016. The points are 30-minute averages of 8-second telemetry measurements sampled every three hours. The *panels* show the temperatures of the front door (*top panel*), front-window mounting ring (*Panel 2*), front-camera electronic box (CEB, *Panel 3*), front CCD (computed from 16-second telemetry), aft optical bench (*Panel 5*), and filter oven (*bottom panel*). Note the different temperature ranges, particularly for the tightly controlled filter oven and nearby optical bench. Annual variations and semi-annual eclipse-season perturbations are visible on the longer term. The first HMI processor reboot occurred on 20 April 2013. The thermal control scheme for elements of the optics package changed on 16 July 2013 and 25 February 2014. Daily differences between Noon and Midnight dominate the short-term variations. Systematic daily variations (see Figure 4) produce what look like multiple lines in the three-hour samples shown here.

Numerous temperature sensors placed throughout the instrument monitor the HMI response to every aspect of its thermal environment (see Appendix B and supplementary material in Schou *et al.*, 2012a, for thermistor locations). Figure 3 shows temperatures at six representative locations in the instrument. Three-hour samples of 30-minute averages of quantities measured every eight seconds highlight long-term variations. The six locations illustrate the variations of different subsystems with varying levels of thermal control: the front door, the mounting ring of the front window, the front-camera electronics box (CEB), the front CCD, the optical bench, and the filter oven. The front window and the last three have the greatest measurable impact on the observables.

The top panel shows the temperature of the front door from 1 March 2010 through the end of 2016. The front door is outside the optics package, and its temperature is essentially uncontrolled, except that it is in thermal contact with other controlled parts of the instrument. There is a jump just before the start of the prime mission in early 2010 when the initial operating temperature was set. The most obvious features are the regular annual variation of about 4 K due to the change in Sun–SDO distance and transient decreases during the twice-annual SDO eclipse season. The instrument was designed to operate near room temperature. The equilibrium temperature has increased by about 9 K since the start of the mission. This is due to changes in reflectance/absorbtion of the front-door surface and to deliberate temperature changes in the nearby front window (see discussion in Section 3.3).

The temperature at the bottom of the front-window mounting ring (temperature sensor 02, TS02), shown in Panel 2, is not directly controlled; instead, the thermistor is attached to the edge of the front window opposite the sensor used to control the temperature. The front-window temperature has been allowed to increase by about 5 K since 2010 in order to keep the focus of the instrument constant. Unlike most other locations, the front-window temperature increases during eclipses because the heaters are turned up to keep thermal gradients in the front window small so that the post-eclipse recovery is shortened (Section 6.2).

The front-camera electonics box (CEB, TS28 in Panel 3) is mounted on the front of the HMI optics package. It also shows variations with annual periodicity (about 3 K) and exhibits short strong dips during eclipses (third panel). The temperature runs a little hotter than most of the optics package because the camera electronics generate heat that is not fully dissipated by its own dedicated radiator. The average CEB temperature increased about two degrees in the first two years, but has been relatively stable thereafter. Shorter-term 24-hour variability is discussed in the next section.

Each CCD detector has its own large radiator on the outboard surface of the instrument that is sheltered from direct solar radiation; it faces solar South (perpendicular to the Sun–spacecraft line) and has a nearly unobstructed view of cold space, except for the Earth. The CCD temperature is kept very low to minimize dark current. The fourth panel shows the temperature at the front CCD detector (TS104, determined from averages of temperature readings made every 16 seconds). The annual variation in temperature is smaller; shorter-term variations dominate. Couvidat *et al.* (2016) determined an intensity sensitivity of 0.25% per degree.

Panel 5 shows a temperature measured on the optical bench inside the optics package (TS23). During the first three years of operation, the temperature was controlled by specifying a specific power input from the internal heaters. The constant overall duty cycle of the heaters was occasionally adjusted, but there was no active on-board control. Consequently, the temperature varied with the overall equilibrium temperature of the instrument, and an annual variation of about 1 K was apparent. On 16 July 2013, the scheme was changed to turn the heaters on at a specified duty cycle only when the temperature goes below a set minimum. Subsequently, the temperature variation has been greatly reduced, and even the response to eclipses has significantly diminished. A consequence of this is discussed in Section 3.3.

The bottom panel of Figure 3 shows the temperature measured on the outside of the tightly controlled filter oven. The oven is kept warmer than the rest of the optics package so that its temperature can be more precisely controlled. The specification for thermal control of the filters is 0.01 K per hour. While the specification is more than met within the oven, an annual peak-to-peak variation of about 0.05 K remained at the externally mounted sensor. On 20 April 2013, the HMI processor was rebooted for the first time, and this eliminated a small amount of current that had been flowing in the redundant oven-thermal-control system. The internal oven temperature did not change, but the temperature measured at the external sensor did because the gradient between the oven and the rest of the instrument was altered. The 16 July 2013 change to the optical-bench thermal-control scheme nearly eliminated the annual variation. Inside the oven, the annual variation was attenuated by a factor of two to three (not shown).

### Short-Term Instrument Temperature Trends

**Figure 4:**
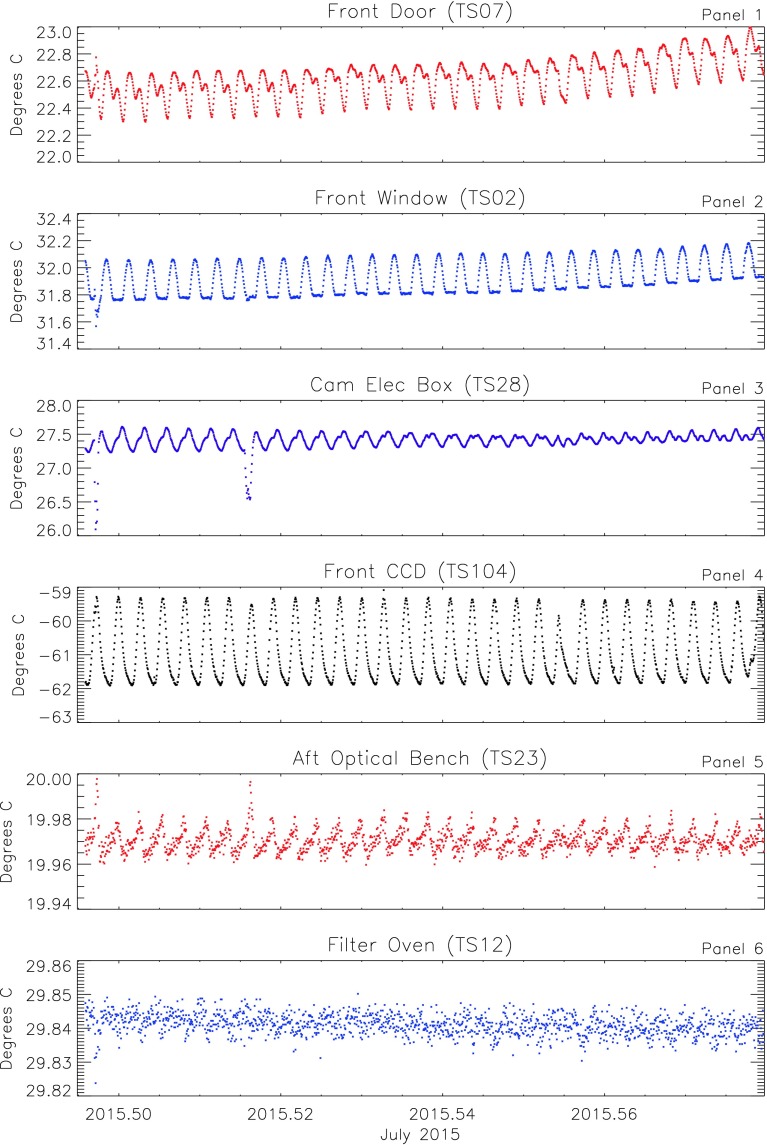
HMI instrument subsystem temperatures for July 2015. Data are 30-minute averages and highlight the daily variations. *Panels* show temperatures for the front door (*top*), front-window mounting ring (*Panel 2*), CEB (*3*), front CCD (*4*), optical bench (*5*), and filter oven (*bottom*). The temperatures of the front door, CCD, and CEB are not actively controlled. The CCD radiators are oriented to see (mostly) dark, cold space. The temperature of the front-window mounting ring at the sensor (TS02) shown in *Panel 2* remains constant during only part of the day. The door and electronics box show more complex daily patterns due to varying exposure to the Earth and other environmental factors.

Figure 4 shows temperatures measured at the same locations in the instrument for July 2015 – after the changes in the temperature control scheme. This month is fairly typical and was selected because it has a few interesting features that can be examined in some greater depth. Averages have been made for 30 minutes (225 eight-second measurements) to highlight shorter-term variations and reduce noise. Unless there is some anomalous event, measurements of variations on timescales shorter than 30 minutes may not be meaningful because the digitization interval (about 0.05 K, depending on gain) and read noise (the standard deviation of five-minute averages is about 0.03 K) are larger than the actual short-term variability in most instrument temperatures.

Because of its $28^{\circ }$ inclined geosynchronous orbit (up to about $52^{\circ }$ to the Ecliptic), the environment of the spacecraft changes with a 24-hour period, and the relative viewing angles of the Earth and Moon at a particular time of day change during the month and year. The orbit was chosen so that the spacecraft remains near $100^{\circ }$ W longitude, in constant view of the ground station in White Sands, NM. Eclipses occur only during the Spring and Fall when the spacecraft passes near the Equator at local Midnight. The eclipse dates change as the orbit slowly precesses.

The top panel of Figure 4 shows daily variations of the uncontrolled front-door temperature (TS07). Short-term temperature variations are dominated by changes in the spacecraft environment, primarily the view of the Earth, and by thermal changes elsewhere in the instrument. The maximum daily temperature occurs shortly after 06 UT, local Midnight at the ground station, when Earth is closest to the Sun–SDO line. A second smaller maximum appears slightly less than 12 hours later in phase with the temperature maximum of the CCD camera (discussed below). The temperature minimum is fairly sharp and occurs near 0 UT, which is dusk at the spacecraft. The daily temperature range is about 0.3 K.

The temperature of the front window is controlled using measurements from a sensor (TS01) located on the mounting ring opposite the one shown in the second panel (TS02). There is a temperature gradient across the front window. During the first half of the day (0 – 12 UT), the Earth is in view of the front window, so it radiates less energy. As a result, the temperature at TS02 rises due to the change in gradient across the window. During the other half of the day, the window cools more efficiently, the gradient changes, and the temperature at TS02 is better regulated. The front door (shown in the top panel) is close to the front window, so it is affected by the thermal control of the front window.

The front-camera electronics box (Panel 3) is mounted on the front of the instrument. It is insulated from direct Sun and has a shield / radiator mounted perpendicular to the Sun–SDO line. Changing views of the Earth affect the amount of heat that is absorbed and also affect the temperatures of other parts of the spacecraft in its field of view. The daily thermal variation of the CEB is more complicated; it shows profile features of both the front window and the CCD (Panel 4).

The CCD temperatures are not actively controlled, but they are kept very cold using independent large radiators mounted on the outboard side of the instrument, ordinarily facing solar South (TS04, shown in Panel 4 of Figure 4). The visibility of the Earth from the radiators changes significantly during the 24-hour orbit, and the daily CCD temperature variation is fairly large: nearly 3 K. The phase of the environmental variation shifts throughout the year. The SDO is located below Earth's Equator at local Noon during one half of the year and above it during the other half; eclipses occur during the transition. In July the fairly sharp daily temperature profile of the CCD peaks at local Noon (about 20 UT) when the Earth is near the anti-sunward direction and most visible to the radiators. Whatever causes the variation in the CCD temperature also affects other external, uncontrolled parts of the instrument, as seen in Panels 1 and 3. Multiple lines appear in the corresponding panels of Figure 3 because of the three-hour sampling of the systematic daily temperature profile.

The optical-bench temperature is controlled using measurements made at a particular location; Panel 5 shows that the temperature measured at a nearby location on the optical bench varies within a range of 0.02 K. The temperature has a sawtooth daily profile and peaks each day at the same time as the CCD detector.

The filter oven is thermally isolated from the rest of the instrument, has a long thermal time constant, and varies in temperature by less than 0.01 K with only a very weak daily pattern (TS12, in the bottom panel). Remaining variations at the surface of the oven shown here are consistent with read noise of the sensors.

There are several interesting features of note during the month. On 1 July and 8 July, there are clear offsets in the front-camera electronics-box temperature (Panel 3) that can also be seen to varying degrees in the optical bench, front window, and front door (Panels 5, 2, and 1, respectively). On 1 July, the SDO performed a “cruciform maneuver” for the purpose of calibrating the EVE instrument. Over the course of about 4.5 hours, the spacecraft was pointed to 112 different locations up to $3.05^{\circ }$ away from the Sun along two orthogonal directions, and this caused small changes in the temperatures. On 8 July, small offpoints of the spacecraft were made to determine AIA and HMI offset flat fields. The corresponding temperature perturbations were smaller.

Careful inspection shows that on 22 July, the front-CCD temperature profile was unusual (Panel 4). Small perturbations in the optical-bench and camera-electronics-box temperatures (Panels 5 and 3) can also be perceived. These occurred during a spacecraft-roll maneuver performed for HMI calibration (see Section 2.4.2). During the roll, the Sun–Earth pointing is maintained, but the spacecraft is oriented with solar North at 16 different roll angles. The change in roll changes the viewing angle of the Earth from the HMI radiators.

### Plate Scale

**Figure 5:**
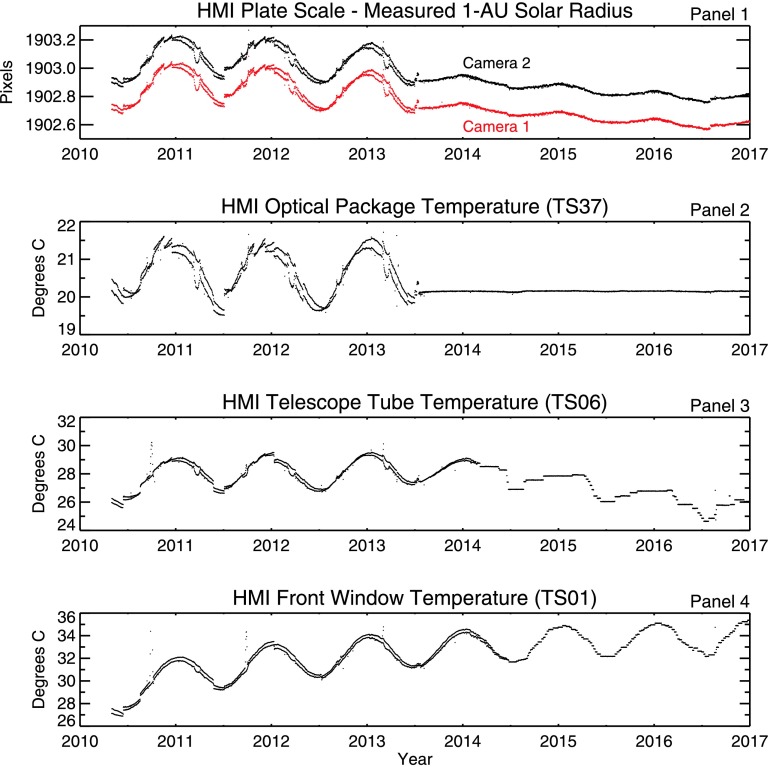
Variation of the HMI plate scale (CDELT1) with time (*top panel*) compared to three different instrument temperatures. The solar radius has already been normalized to 1 AU using known geometric parameters. Camera 2 is shown in *black*, the slightly cooler Camera 1 is plotted in *red*. The *second panel* shows the temperature measured by a representative temperature sensor (TS37) in the HMI optics package. *Panel 3* shows the temperature of the telescope tube. The *bottom panel* shows the front-window temperature. In each *panel* two values are shown for each day, one measured near the orbital perihelion, and the other near aphelion. These values roughly correspond to daily extremes in the instrument temperature.

The plate scale is set by the mechanical and optical properties of the telescope and is measured by determining the observed radius of the solar image in CCD pixels and applying a geometric correction to normalize the value to 1 AU. The HMI plate scale correlates strongly with the temperature of the HMI optics package and to a lesser degree with the telescope-tube temperature, as shown in Figure 5.

The pronounced annual periodicity present during the first three years is due to temperature drift of the HMI instrument caused by the change in irradiance that is due to variation in the Sun–spacecraft distance. Daily variations are driven primarily by changes in the spacecraft environment related to the SDO orbit.

In the early years, when the instrument temperature varied by slightly more than a degree during the course of a year, the measured radius varied by about 0.3 pixels (0.15 arc seconds). As described in Section 3.1, the temperature-control scheme for the optics package was changed on 16 July 2013 to reduce variations in the temperature. The variations in plate scale were greatly reduced. Similar changes were made to the temperature-control scheme for the telescope tube and front window on 25 February 2014. Since then, more frequent temperature adjustments have been made to keep the focus of the instrument in the proper range. The gradual long-term decrease in the measured solar radius may be related to changes in the front-window temperature (which affects magnification), tube temperature (which affects the distance between lens and image), or other factors.

Using HMI data collected during the 2012 Venus transit, Emilio *et al.* (2015) derived a 1 AU solar radius in the continuum wing of the line of $959.57 \pm 0.02$ arcseconds, equivalent to $695,946 \pm 15\mbox{ km}$. Similarly, Couvidat *et al.* (2016) found that the image of the Sun is slightly larger than expected. For the image scale, the ratio of their best estimate to that in the headers is 0.99992053. Consequently, we conclude that for the HMI spectral line, the reference radius of the Sun (keyword RSUN_REF) should be decreased by about 55 km to 695,944,685 m.

## Optics and Filter Issues

This section describes calibrations and observations made to assess the optical performance of the HMI instrument and elements of the filter system. A more complete discussion of the filter calibration is found in Couvidat *et al.* (2016).

### Instrument Throughput Changes

**Figure 6:**
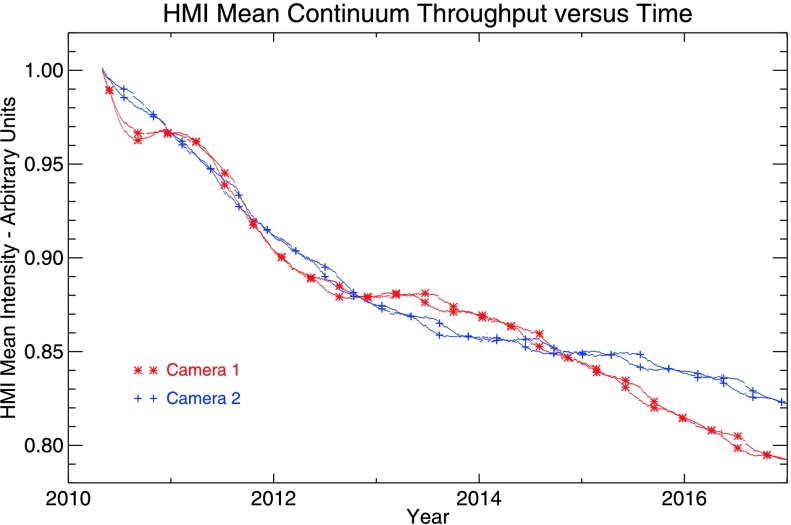
Evolution of the end-to-end instrument throughput during the SDO mission. The average on-disk solar continuum intensity measured with Camera 1 (Camera 2) is plotted as a function of time in *red* (*blue*). The throughput of Camera 1 had decreased by slightly more than 20% by the end of 2016. The continuum intensity is measured during the twice-daily calibration sequences at about 06 UT and 18 UT. *Symbols* highlight 06 UT and 18 UT measurements approximately every 200 days for each camera. Short-term differences in a single camera primarily reflect temperature changes that are due to solar-irradiance and thermal-environment variations. Values, normalized to the intensity of the first image, have been corrected for the Sun–SDO distance and exposure time. Values have also been empirically adjusted to compensate for a permanent change in image crop radius on 28 January 2015.

The instrument throughput has been slowly decreasing since launch. Figure 6 shows the average solar intensity measured in twice-daily full-disk continuum exposures ($\mbox{Frame ID} = 10{,}000$) for each camera. The DATAMEAN values have been corrected for exposure time, the Sun–SDO distance, and for a one-time change in the image crop radius at 19:51 UT on 28 January 2015. The exponentially decreasing decay rate observed in both cameras is generally consistent with expected effects of radiation damage darkening the front window. Short-term variations of a single camera or between the cameras is likely due to the changing thermal environment. Couvidat *et al.* (2016) measured a temperature sensitivity of $-0.25\%$ per degree in Camera 2, but as shown in Panel 4 of Figure 3, except for regular daily and annual changes, the nominal temperature measured near the CCD has not changed much over the course of the mission. The origins of the long-term differences between the two cameras are not understood. The local-Noon–Midnight asymmetry (6 – 18 UT) is greatest in the middle of the year when Earth is south of the SDO and thus most visible to the radiators at local Midnight.

The gradual decrease in instrument throughput requires occasional exposure-time increases to maintain a roughly uniform signal intensity. Since launch, the exposure duration has been increased three times, in each instance by 5 ms, as shown in Table 3. There is still sufficient margin in the timing of the camera image taking to compensate for further throughput decreases; the current mode of operation allows for exposures of up to 430 ms without compromising the basic 45-second cadence.

**Table 3:** HMI camera exposure-time adjustments

Date	Front camera (1)	Side camera (2)
01 May 2010	125 ms	115 ms
13 Jul 2011	130 ms	120 ms
16 Jan 2013	135 ms	125 ms
15 Jan 2015	140 ms	130 ms

HMI observables are computed from sums and differences of filtergrams, so exposure-time uncertainty contributes directly to errors in the measured quantities. A mechanical shutter motor controls the exposure time by rotating the cut-out sector of an opaque disk into place, with a pause in the *open* position for a specified time. The shutter is located in the observing beam near an image of the pupil when in Obsmode. The mechanical exposure time can be specified with precision of about 120 microseconds and has an observed standard deviation of 13.2 microseconds, about a part in 10,000 of the nominal exposure. The difference between the commanded and actual exposure time is determined with precision of one microsecond and accuracy better than 4 microseconds using integral detectors to determine the precise times that the leading and trailing edges of the open sector rotate past each of three characteristic locations in the beam. The actual exposure time is used in the analysis. Typical exposures are 115 – 140 milliseconds. The 4-microsecond exposure-time knowledge is a part in 30,000 of the nominal exposure time. This is a factor of three or more better than what is required to beat the photon noise level for global averages of the mean magnetic field and the large-scale velocity for low-spatial-degree helioseismology. The SDO/HMI exposure time is monitored far more closely than it was for the *Solar and Heliospheric Observatory/Michelson Doppler Imager* (SOHO/MDI: Scherrer *et al.*, 1995) and has much less variability. See Appendix B for a plot of the mechanical-exposure quality.

### Distortion

**Figure 7:**
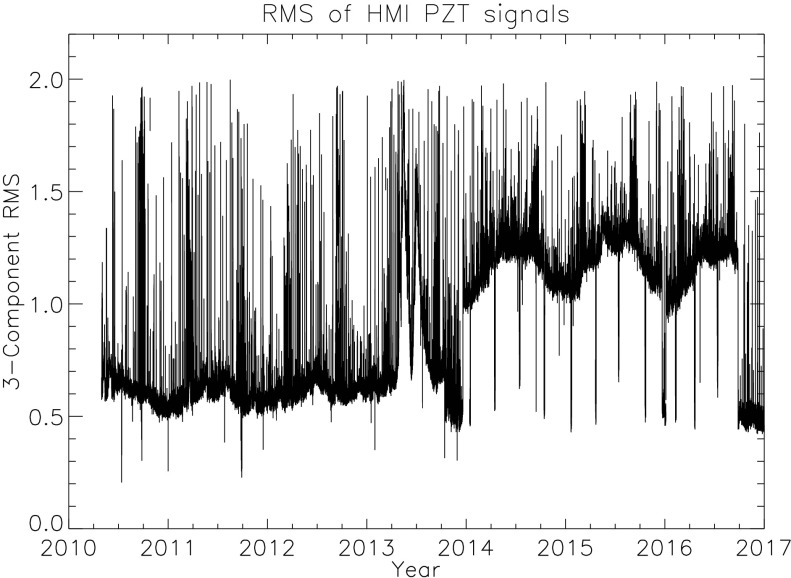
Voltage variations of the image stabilization system (ISS) *versus* time. The HMI uses three PZTs to control the guiding mirror based on an error signal determined by limb sensors. The RMS of the voltage over an hour is an indication of the pointing jitter for which the system must compensate. The *plot* shows the RMS of the three one-hour-RMS values *versus* time. The SDO pointing was fairly stable until mid-2013, when the performance of one of three inertial reference units (IRU) started to deteriorate. A new mode using just two IRUs commenced in October 2013. The operating temperature of the IRU wheels was changed in September 2016, and the spacecraft pointing stability improved noticeably. For clarity, values outside the range 0.2 – 2.0 are omitted.

Image distortion arises because of small imperfections in the optics, including the optics that move to tune the instrument. The distortion map determined prior to launch for each camera (see Figures 7 and 8 of Wachter *et al.*, 2012) has been characterized using Zernike polynomials. The fitted instrumental-distortion correction is applied to each Level-1 filtergram. The maximum displacement before correction is less than 2 pixels and occurs near the top and bottom of the CCD camera; the mean residual distortion after correction is $0.043 \pm 0.005$ pixels. Differences between the front and side cameras are on the order of 0.2 pixels. Couvidat *et al.* (2016) analyzed HMI images taken during the Venus transit of 6 June 2012 and found that all along the path of the planet, the distortion-corrected observed position agreed with the ephemeris coordinates to better than 0.1 pixels (0.05 arcseconds).

### P-Angle

The roll angle of the solar image relative to the instrument is commonly called the p-angle (not to be confused with the position angle determined for Earth-based observations). In the case of HMI, the top of the CCD is nominally near the solar South Pole, so the WCS standard CROTA2 keyword that gives the angle between heliographic north and CCD coordinates typically has a value very close to $180^{\circ }$. For the HMI, the p-angle $= 180 - \mathsf{CROTA2}$.

Couvidat *et al.* (2016) reported on a careful analysis of both the absolute p-angle based on observations of the 6 June 2012 Venus transit and the relative p-angle of the two cameras based on comparison of near-simultaneous images obtained by the two cameras in July 2012. They find that the p-angle for the front-camera is $-0.0135 ^{\circ }$ and for the side camera $+0.0702 ^{\circ }$. The difference in p-angle between the two cameras is $0.0837 ^{\circ }$, with a constant drift rate of $-0.00020 ^{\circ }$ year^−1^ during the SDO prime mission. The drift is probably due to curing of materials used to mount the CCDs or to thermal changes.

The absolute p-angle was also determined by Liang *et al.* (2017) for the Mercury transit using the same methods as were used by Couvidat *et al.* (2016). However, the much smaller size of Mercury meant that no annulus extraction was done. They found that the values for Camera 1 changed from −0.0140 to −0.0114 ($+0.0026$) and those from Camera 2 from $+0.0712$ to $+0.0735$ ($+0.0023$). Given the size of the residuals seen by Couvidat *et al.* (2016), the difference does not appear to be significant.

### Camera Differences

The front and side cameras of HMI are not identical, and their images exhibit slightly different properties, for example in their focus, alignment, and the occurrence of bad pixels. Of course, the temperature and radiation environments of the two cameras also differ to some degree. Although the CCD radiators are adjacent and on the same solar-south-facing side of the instrument, the radiators for the camera-electronics packages have different geometries. The only significant differences in the optical paths are due to a beam splitter, fold mirrors, and shutters that direct the light to the two cameras after all of the other optics. Since 13 April 2016, filtergrams from the two cameras have been combined to compute the vector magnetic field (Hoeksema *et al.*, 2014; Couvidat *et al.*, 2016). Figure 2 shows that there is only a small drift in focus difference between the two cameras during the lifetime of the mission, probably due to aging of materials that affect the CCD mounting position or to thermal drifts.

### ISS Performance

Basic spacecraft pointing information is provided by three inertial reference units (IRUs). The spacecraft relies on signals from AIA for more fine-guiding information. Small, rapid pointing variations are driven by movements of mechanisms throughout the spacecraft. The HMI image-stabilization system (ISS) uses a tip–tilt mirror to remove fine-scale jitter measured at a primary image plane in the instrument. The ISS measures the solar-limb position using four orthogonal detectors to sense image motion on the limb. The HMI guiding mirror has a three-point PZT actuator to compensate for position errors in the observed limb position. The ISS holds the image location constant to about 0.025 arcseconds (a twentieth of a pixel) with a frequency roll-off of a factor of two at about 50 Hz (Schou *et al.*, 2012a). The PZTs nominally operate at about 35 V, and there is a superposed annual period of amplitude about 5 – 10 V associated with variations in the spacecraft thermal environment and size of the solar image. The nominal set point can also change when the instrument legs are moved to recenter the image (approximately monthly).

The RMS voltage variation for each PZT computed over an hour is on the order of half a volt, with occasional spikes when spacecraft mechanisms are active. The RMS value of the three computed PZT-RMS values is an indicator of the magnitude of the jitter signal. Figure 7 shows the hour-averaged three-PZT RMS value of the ISS voltages from 1 May 2010 to the end of 2016.

Regular large-amplitude spikes are due to brief weekly and bi-weekly excursions when the instrument is intentionally pointed away from Sun center for calibrations. Regular intervals of increased RMS are also visible each Spring and Fall during eclipse season. The ISS control loop is ordinarily turned off around eclipse times and during spacecraft off points.

The SDO is equipped with three IRUs to provide information to help keep the solar pointing stable; however, the operation of the IRUs has changed during the mission. The IRUs were operated at a temperature that was colder than optimal during most of the mission because of concerns about potentially deleterious effects of their heaters on the spacecraft battery. As a result, some jitter was introduced by the wheels. In 2013, the performance of IRU-1 began to deteriorate more rapidly, and on 12 October 2013, the current draw increased sharply. The next day, IRU-1 was removed from the control loop, and it was powered down in December 2013. Since that time, SDO has operated with only two IRUs. In early 2015, IRU-2 exhibited early signs of similar behavior. A test in late 2015 showed that increasing the IRU temperature eliminated the worrying symptoms of IRU-2 and improved overall jitter levels. After careful analysis of the effects on the battery, the IRU temperatures were raised on 16 September 2016. The decrease in the jitter signal is apparent in Figure 7. These changes in operation of the spacecraft IRU units have had no apparent effect on the final performance of the ISS system, nor have they been detected in the HMI science products, except for an increase in five-minute power in the full-disk intensity means between October 2013 and September 2016 (R. Howe, private communication 2016) and in local-correlation-tracking results (B. Löptien, private communication, 2015) that may be due to jitter in the spacecraft roll angle.

### HMI Filter Element Wavelength Drift and Tuning Changes

The HMI uses a series of filters to select the wavelength of each filtergram. The entrance window and broad-band blocking filter are followed by a five-stage Lyot filter and two Michelson interferometers. The final stage of the Lyot (E1) and the Michelsons are tuneable. The nominal wavelength of each tuneable element is set by rotating a half-wave plate. Rotation of the wave plate by $90^{\circ }$ scans the element through its free spectral range (FSR). For convenience, the wavelength tuning is characterized in terms of the phase within the FSR. This means that scanning $360^{\circ }$ in phase tunes through the entire spectral range of the element, so each $1.5^{ \circ }$ step of the hollow-core motor that holds the wave plate changes the phase by six degrees.

**Figure 8:**
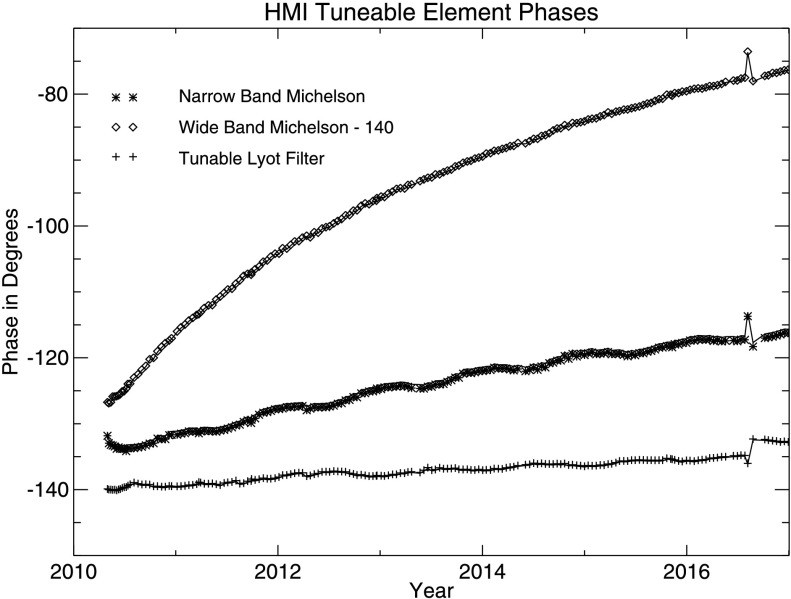
Wavelength drift of the HMI tunable elements determined during regularly scheduled detunes. The phase for each element has an arbitrary zero, and $360^{\circ }$ corresponds to the full FSR of the element. The tuneable Lyot element (*plusses*) drifts slowly with time. The narrowband (NB) Michelson (*asterisks*) drifts only slightly more rapidly. The wideband Michelson (*diamonds*, offset in the plot by $-140^{\circ }$) has the largest drift, about an eighth of an FSR during the mission. A spacecraft anomaly on 2 August 2016 resulted in an extended loss of thermal control that had lasting effects, particularly on the Lyot filter phase. *Symbols* show the fit determined with images from Camera 2, and the connected solid lines show Camera 1; the difference is very small. A handful of anomalous fits are not shown.

The central wavelengths of the filter elements drift with time. The wavelength of each of the three tuneable elements can be determined from the bi-weekly detune calibration sequences described in Section 2.3. A relative minimum in intensity occurs when an element is tuned to the spectral-line center. The average phases of the HMI tunable elements change slowly with time, as can be seen in Figure 8. No correction has been made for the motion of the spacecraft since the detunes are ordinarily taken when the Sun–SDO velocity is small.

It is important to cotune the filter elements to the same wavelength and to keep the wavelength range over which the filtergrams are taken centered on the Fe i spectral line. The observed drifts warrant regular retuning of the instrument. The wideband (WB) Michelson exhibits a stronger time-dependence, whose origin is thought to be the glue holding the mirrors in the two legs; it is believed that the glue in the vacuum leg has expanded or contracted with time. A similar issue was encountered by SOHO/MDI. The rate of change in the WB Michelson phase is slowing down. The instrument tuning has been adjusted about once per year, as indicated in Table 4.^1^ The table also indicates the wavelength tuning ID number (WTID) and the specific index positions of the three tuning motors.

**Table 4:** Dates of HMI retunings.

Retuning date and TAI time	Wavelength tuning ID (WTID)	Reference tuning position		
		Lyot/E1	Wideband	Narrowband
30 Apr 2010 22:24	10	36	58	82
13 Dec 2010 19:45	11	37	56	82
13 Jul 2011 18:35	14	37	54	82
18 Jan 2012 18:15	17	37	53	81
14 Mar 2013 06:42	20	37	52	81
15 Jan 2014 19:13	23	37	51	80
08 Apr 2015 18:51	26	37	50	80
27 Apr 2016 18:56	29	37	50	79
19 Apr 2017 19:58	31	38	49	79

**Figure 9:**
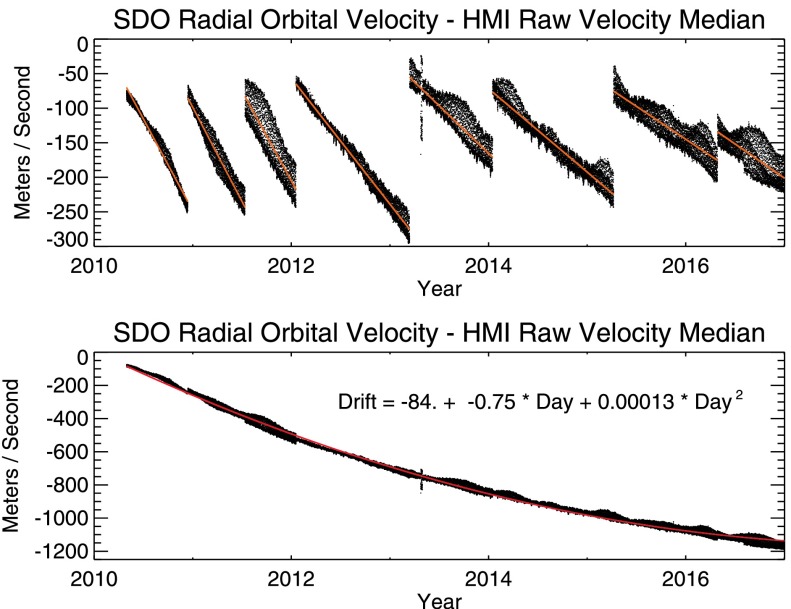
Velocity drift of the HMI observable. The *top panel* shows the difference between the known Sun–SDO velocity and the median uncorrected velocity determined from an HMI Dopplergram. The drift in the measured velocity is due to the drift of the HMI filter elements. Breaks in the curve occur when the filter tuning is changed. The *bottom panel* shows the same, but without the velocity offset due to the retuning. A polynomial fit to the velocity drift is given, which indicates that the drift was initially slowing by −0.75 m s^−1^ per day.

If the instrument were tuned and calibrated perfectly, the measured median velocity of the Sun would be nearly the same as the Sun–SDO velocity. Figure 9 plots the difference between these two quantities, demonstrating the effect of the slowly changing wavelength and the effects of compensating changes in the HMI filter tuning. The Sun–SDO velocity is known to a few mm s^−1^ and the baseline zero offset is due to the nominal tuning of the instrument. The daily scatter is due to the effects of changes in the instrument environment and to actual solar signals that appear in the median-velocity signal. Changes in the short-term noise level arise from changes in sensitivity and imperfections in calibration discussed elsewhere. The upper panel shows that the residual velocity decreases with time at a significant rate and that the rate seems to slow with time. The tuning has been adjusted regularly to keep the offset from zero less than about 300 m s^−1^. The bottom panel adds back in the velocity offset due to the changes in the tuning, as determined by matching the endpoints of the linear fit for each subset. A quadratic fit matches the curve very well and shows that the overall drift in meters per second is $-84 - 0.75 D + 0.00013 D^{2}$ for $D$ measured in days from the start of the prime mission.

The constant and evolving spatial characteristics of the HMI filter elements are described in considerable detail by Couvidat *et al.* (2016) and Couvidat *et al.* (2012b).

## Level-1 Corrections: Camera and Detector

The data capture system (DCS) at Stanford's Joint Science Operations Center (JSOC) receives raw science data directly from the SDO ground station; housekeeping and other spacecraft data come via the mission operation center at NASA/Goddard. The image data are extracted, combined with the appropriate metadata, and packaged as image files. These raw, uncorrected filtergrams are referred to as Level-0 data, and they are typically available within three minutes of the image acquisition onboard the spacecraft. The first stage of data processing applied to these images at the JSOC, which includes overscan row removal, dark-current and flat-field correction, and cosmic-ray detection, as well as added metadata, generates Level-1 data. This processing is done twice: once as quickly as possible to generate the near-real-time (NRT) data for use in space-weather applications, and then a second time, typically four days later, with occasional ground-based transmission gaps filled and with better calibrations to generate the definitive Level-1 data. The Level-1 processing is described in this section.

### Dark-Current Correction

Dark frames are taken with each camera twice a day as part of the calibration sequences started at 06:00 UT and 18:00 UT. Zero-length pedestal-current (bias) measurements are not taken; the CCD bias and dark current are measured together, and we do not distinguish between them. The measured dark current in both cameras has been extremely stable over the course of the mission, with average dark values of 122 counts and 131 counts for Cameras 1 and 2, respectively. To minimize the impact of photon noise on the dark correction, average dark frames are generated from the individual darks every three months, and these averages are used in the Level-1 processing. There is a diurnal variation in the temperatures of the CCDs that likely gives rise to a small variation in CCD dark signal, but this is not currently measured or corrected for. In principle, data from the overscan area could provide additional information about dark current and other parameters for each image.

### Flat Field Correction

**Figure 10:**
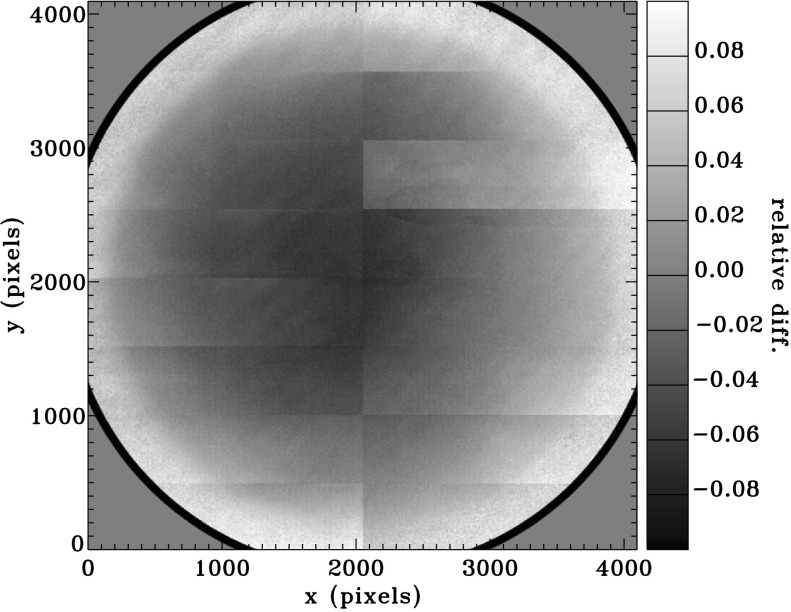
Relative differences between a flat field from 23 January 2015 and one from 1 March 2010. Both flat fields are for Camera 2.

Pixel-to-pixel gain variations in the CCD detectors are corrected for using flat fields measured for each camera. Because there is no way to illuminate the CCDs on orbit with a sufficiently uniform light source, the pixel gains are determined by shifting the solar image to various locations on the CCDs. The procedure for using these images to determine the flat field is described by Kuhn, Lin, and Loranz (1991), Toussaint, Harvey, and Toussaint (2003), and Wachter *et al.* (2012). The solar image can be shifted in two ways, and both are used in determining HMI flat fields. First, the entire spacecraft can be slewed to a set of off-points. This is done quarterly, and it involves nine off-point positions in a cruciform pattern. The entire maneuver takes approximately two hours and forty minutes. The second method uses the instrument ISS to shift the image. The PZTs in the ISS are activated to tilt the ISS mirror to a predetermined set of offsets. PZT flat fields are performed weekly to provide a good measure of small-spatial-scale sensitivity, whereas the quarterly offpoints provide a better large-scale flat field. The flat fields of both cameras have evolved slowly over the course of the mission. The difference between the front-camera flat field at the beginning and end of the prime mission is shown in Figure 10.

A different method of generating flat fields, using the rotation of the Sun to smooth out inhomogeneities in the solar image, has also been implemented. The algorithm used to calculate rotational flat fields is described by Wachter and Schou (2009). Rotational flat fields are expensive to compute and are not used in the current Level-1 HMI data, since they provide only a small improvement over the PZT method.

### Bad Pixels and Cosmic Rays

Each filtergram taken by HMI has a number of bad pixels that must be identified and properly treated. There are a very small number of totally bad pixels: none in Camera 1 and just three in Camera 2. In addition, pixels from the quarterly off-point flat fields with gains less than 50% of the average gain are considered to be permanently bad and are identified as such in each filtergram. The list of such pixels is propagated into each Level-1 filtergram record. Camera 1 has 45 pixels flagged as permanently bad, and this has been consistent since the beginning of science operations. The number of bad pixels in Camera 2 increased from 31 to 34 over the course of the prime mission. As with Camera 1, pixels flagged as bad are consistent from off-point to off-point.

Transient events (cosmic rays) account for the remainder of the bad pixels in each filtergram. Cosmic-ray hits are first detected by applying a high-pass filter to each filtergram and flagging pixels that exceed a certain threshold. In the production code, this threshold is 10.5 times the variance in the center of the image. These pixels are included in the Level-1 bad-pixel list. Cosmic rays are detected out to 0.98 of the solar radius, even though image statistics are computed to 0.99. This may be adjusted in the near future.

A second cosmic-ray-detection algorithm is employed after individual Level-1 filtergrams are generated. Run daily as part of the rotational flat-field module, the algorithm identifies bad pixels in tracked locations based on intensity variance over about 20 minutes. False identifications in the initial single-filtergram detection algorithm are sometimes found. The results for each image are logged, but they are not easy to recover. The higher-level processing modules that combine multiple filtergrams to calculate the observables (Couvidat *et al.*, 2016) exclude the bad pixels from the temporal and spatial interpolation. This second cosmic-ray detection is not run for HMI-NRT observables.

**Figure 11:**
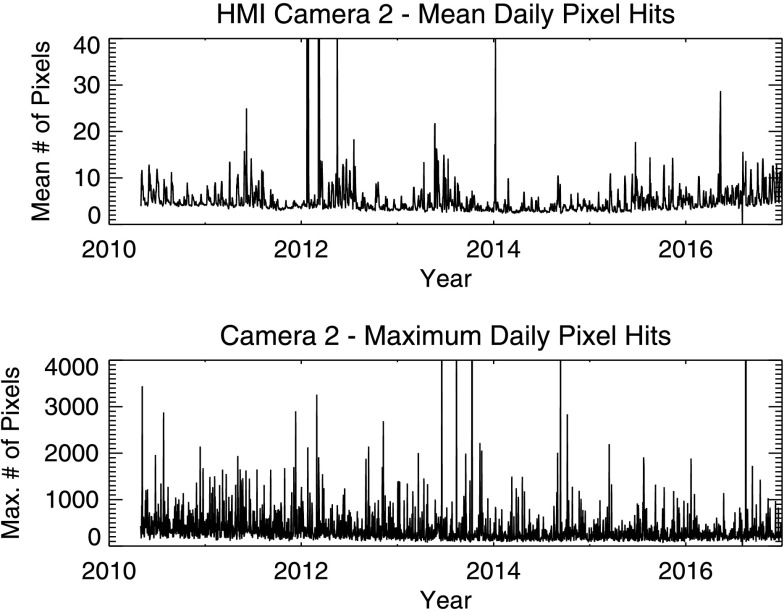
Daily mean and maximum number of bad pixels per image as a function of time for Camera 2.

The number of pixels removed due to cosmic rays varies throughout the year and with solar activity. Figure 11 shows the daily mean and maximum number of pixel hits in Camera 2. Camera 2, mounted on the Sun-facing side of the instrument, generally takes roughly twice as many hits as the other camera.

### Solar-Radius Correction for Height of Formation

**Figure 12:**
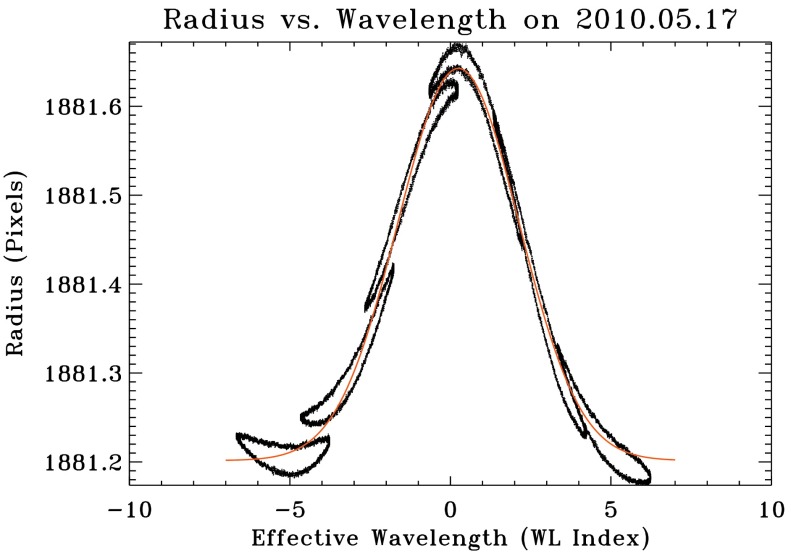
Solar radius returned by the limb finder as a function of the effective wavelength at which the image is taken. Each of the *six closed loops* shows the radius determined for a particular tuning of the HMI wavelength filter system over the course of 17 May 2010, as the solar line shifts relative to HMI during the orbit. The hysteresis arises because of temperature changes in the instrument correlated with orbital position. The *solid line* is the Gaussian fit described in the text computed for this particular day.

The height of formation near the 6173 Å Fe i spectral line changes with wavelength by a few hundred kilometers (Fleck, Couvidat, and Straus, 2011; Emilio *et al.*, 2015). Because the standard HMI observing sequence samples the solar Fe i line at six wavelengths separated by about $68.8\mbox{ m}$Å, the apparent size of the Sun varies with wavelength by as much as half a pixel. Figure 12 shows the measured solar radius as a function of the wavelength index, where each index step corresponds to a nominal $34.4\mbox{ m}$Å HMI tuning-motor increment relative to line center.

Even though the location of the solar limb depends on wavelength, the physical scale of the image does not change. To account for this properly, the radius returned by the limb finder is adjusted for use later in the processing pipeline when filtergrams are resized. Specifically, the values returned by the limb finder (X0_LF, Y0_LF, and RSUN_LF) are corrected for the wavelength dependence in the keywords CRPIX1, CRPIX2, and R_SUN.

The limb-finder radius is reduced by a wavelength-dependent quantity

1 $$ \Delta R = A \exp\bigl(-(wl_{x} - wl_{0})^{2}/wl_{w} \bigr), $$

where $wl_{x}=wl-\mathsf{OBS\_VR}/\mathrm{d}v\,\mathrm{d}w$, $wl$ is the integer wavelength index of the image relative to the index of the center wavelength, OBS_VR is the known Sun–SDO radial velocity, and dvdw = $\delta \lambda / \lambda \times c = 0.0344 / 6173.3433 \times 299792458$. The values of $A$, $wl_{0}$, and $wl_{w}$ are the result of a Gaussian fit to the solar radii returned by the limb-finder as a function of the wavelength position of the images.

**Figure 13:**
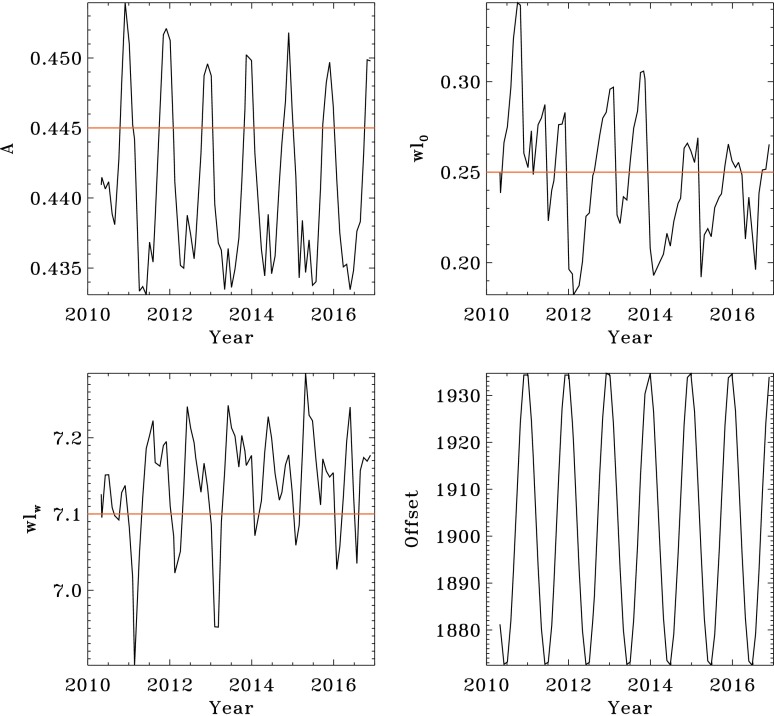
Variation with time of the Gaussian-fit parameters that characterize the height-of-formation correction. The *upper-left panel* is the scaling factor [$A$]. The *upper-right panel* shows $wl_{0}$; the *lower-left* is $wl_{w}$; and the *lower-right* is the offset due to distance (not used in the correction). Eighty one-day fits are shown for the months from May 2010 through December 2016. The standard values are indicated by the *horizontal red lines*. See text for details.

The radius–wavelength relation varies somewhat from day to day depending on average velocity and the instrument environment. Figure 13 shows the observed temporal dependence of the three fitted parameters as well as the baseline offset due to Sun–spacecraft distance. The observables pipeline code uses the following standard values: $A=0.445$, $wl_{0}=0.25$, and $wl_{w}=7.1$. The standard value of $A$ appears in the plot to be too high by as much as 0.005 arcseconds (about 35 km), a significant fraction of the 55 km error in the reference solar radius RSUN_REF discussed in Section 3.3.

A single radius and center-position correction is made for each filtergram, but of course the velocity due to solar rotation also shifts the nominal line position by a comparable amount. This east–west antisymmetric wavelength shift causes an additional position-angle-dependent radius change and image offset for which no correction is made.

### Additional Metadata

Level-1 filtergrams are associated with a variety of metadata stored as keywords in the JSOC database. Information about the status of the instrument from both spacecraft telemetry and the science data streams is associated with the Level-0 filtergrams, and the relevant data are propagated through to Level 1. The Level-1 processing adds information about the spacecraft state, location, and pointing, as well as image scale and centering. Information on spacecraft position and velocity are obtained from spacecraft ephemeris data provided by the flight operations team. Image coordinate information follows the WCS standard (Greisen and Calabretta, 2002) and is computed from a combination of a fit to the solar limb and the spacecraft-ephemeris information. Keywords set in the Level-1 code are listed in Appendix F Table 16.

In addition to these metadata, two keywords are set for the Level-1 filtergrams that deserve somewhat closer attention: QUALITY and CALVER**.

#### Image Quality and the QUALITY Keywords

While nearly all filtergrams taken by HMI over the course of the mission are of nominal quality and suitable for scientific studies, a few are taken under non-nominal conditions, are of degraded quality, or are completely missing. The quality of each filtergram is indicated to the end user by a set of flags stored bit-wise in a 32-bit integer named QUALITY. At Level 0 a QUALITY bit is set when an error occurs in the data transmission and capture, or as a result of certain errors from the instrument. Table 15 in Appendix E describes the Level-0 QUALITY bit masks and meanings. This keyword is propagated to the Level-1 records as QUALLEV0.

At Level 1, a new QUALITY keyword is defined. The bit mask for each flag and its meaning is shown in Table 17 in Appendix G. Nominal science-quality filtergrams have no flags set in the QUALITY keyword, and thus the value will be zero. The most common reason for a non-zero QUALITY is that the filtergram was taken as part of a daily or weekly calibration. In fact, many such filtergrams are no different than those taken in the regular observing sequence and can be used without concern for computing higher-level HMI observables.

The most common flag indicating a degraded filtergram is the ISS-loop-open flag, which indicates that the HMI image-stabilization system is not correcting for image jitter. This occurs during certain calibration sequences and updates of the instrument configuration, but is most often due to the spacecraft not being in its fine-guidance, or “science” mode. This is indicated by the ACS_MODE flag, and is usually due to spacecraft maneuvers or lunar or Earth transits. Another QUALITY bit is set to indicate that the instrument is in thermal recovery after a lunar or Earth transit; for a discussion of these intervals see Section 6.2.

Bits in the QUALITY keyword can also indicate missing metadata or filtergram data. These are mostly due to occasional data corruption that occurs in the instrument electronics; see Section 6.4.

In fact, determining what constitutes a good measurement depends on the use to which the observation is put. The basic quality information for higher level products, *e.g.* Dopplergrams or magnetograms that are computed from multiple filtergrams, is also indicated in an observables-level QUALITY keyword. These are listed in Tables 18, 19, and 20 in Appendix H.

#### Calibration Version and the CALVER** Keywords

Changes to the instrument observing sequence, processing software, and calibration constants, which we refer to collectively as the “calibration version,” are rarely made, but each Level-1 filtergram includes a keyword, CALVER32, that identifies the calibration version used to generate the data. A longer keyword, CALVER64, is used by higher-level data products to convey similar information. Unlike the QUALITY keywords, the CALVER** keywords use nibbles, or 4-bit fields, to denote various calibration changes. The meaning of each field is shown in Table 5. Currently, seven fields are defined; more can be employed if and when new changes are introduced into the processing of HMI data. For Level-1 data, only two of the fields are used: the height-of-formation-correction version, and the instrument-rotation-parameter version. For all currently available Level-1 data, the height-of-formation correction is version HFCORRVR=0x02, and the version number of the rotation parameter, which was corrected after the 11 May 2012 Venus transit, is CROTA2VR=0x01.

**Table 5:** Key to values of the CALVER** keyword nibbles.

Field	Bits	Mask	Name	Note
0	0 – 3	0x0F	HFCORRVR	Height-of-formation code version used.
1	4 – 7	0xF0	CROTA2VR	Version of CROTA2 in the master pointing table.
2	8 – 11	0xF00	N/A	If >0: smooth look-up tables were used.
3	12 – 15	0xF000	N/A	If >0: a non-linearity correction was applied.
4	16 – 19	0xF0000	FRAMELST	If 0x0: Mod C; if 0x4: Mod L; if 0x2 or 0x3: incorrectly processed Mod L.
5	20 – 23	0xF00000	N/A	If >0: PSF/scattered light deconvolution applied.
6	24 – 27	0xF000000	N/A	If >0: rotational flat field used.

## Significant Events and Anomalies

Through the prime mission, the HMI production of nominal science data was more than 95% complete. This section discusses the remaining 5%: the events and anomalies that take place both routinely and unexpectedly that degrade or interrupt science data from HMI. The vast majority of these events are expected and planned for. The semi-annual series of Earth eclipses, as well as occasional lunar transits, obscure the HMI view of the Sun. After eclipses, the most common interruptions are caused by planned calibration sequences that are used to ensure that calibration of HMI science data products continues to be as precise as possible; these are described in Section 2. Science-quality observations are also interrupted during spacecraft maneuvers, which are undertaken for instrument calibrations and for maintaining orbit and control.

On rare occasions, data are lost due to unexpected failures in the instrument, spacecraft, or ground systems. These anomalies are also discussed in this section. Fortunately, all of the data-impacting anomalies encountered were recovered from fully without subsequent adverse effect on instrument health or data quality.

There are four basic ways in which HMI data quality can be affected. First, filtergrams can be taken that are not a part of the standard observing sequence; they are generally not used in generating the science data products. Second, images may be of degraded quality, due to the Sun not being centered, the stabilization system not being on, the instrument being out of nominal focus or temperature range, and so on. Third, image data or metadata may be corrupted, and finally, the data may be missing entirely.

### Spacecraft Maneuvers

The SDO spacecraft periodically performs maneuvers that interrupt HMI science-quality data. Many of these maneuvers are for instrument calibration: eight yearly off-point maneuvers for the EVE instrument, quarterly off-points for AIA and HMI flat fields, quarterly rolls for HMI image-quality monitoring, and quarterly maneuvers to calibrate the AIA guide telescopes (these are used for SDO fine-guidance). In addition to these regular maneuvers, there have been a few special maneuvers: twice to observe the star Regulus for calibration, on 23 August 2010 and 23 August 2011, and for observations of comets Lovejoy and ISON on 15 December 2011 and 28 November 2013, respectively. The spacecraft must also periodically perform burns of its propulsion system for maintenance of its orbit. These station-keeping maneuvers were performed 11 times during the prime mission. Finally, angular momentum must periodically be dumped from the reaction wheels by using the reaction control system (RCS) thrusters. This was done 21 times during the prime mission. Momentum management maneuvers take roughly 14 minutes; station-keeping maneuvers ordinarily take 35 minutes. When possible, maneuvers are performed together to minimize the number of gaps. An events table can be found at aia.lmsal.com/public/sdo_spacecraft_events.txt.

### Earth Eclipses

**Figure 14:**
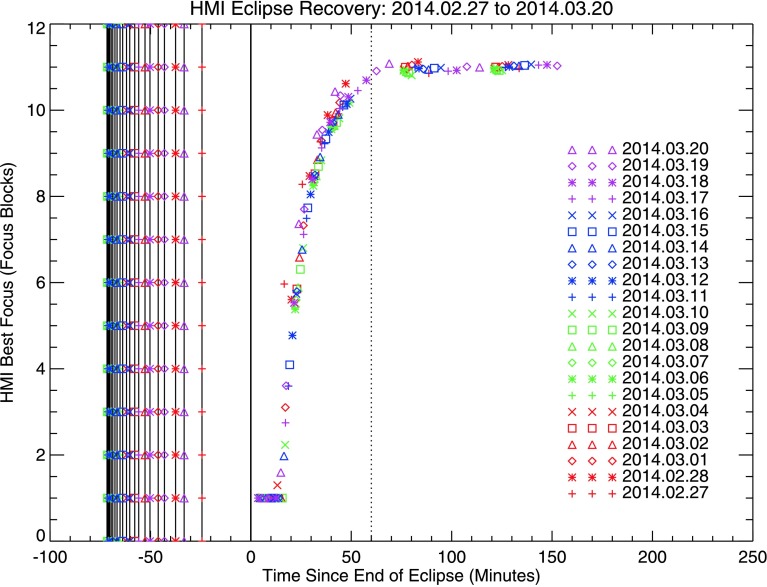
HMI post-eclipse focus recovery during the Spring 2014 eclipse season.

Twice yearly, in Spring and Fall, the SDO view of the Sun is obscured by a series of Earth eclipses. There are between 22 and 24 such daily eclipses per season, occurring near local Midnight of the SDO orbit around 06 UT, and they last up to 72 minutes. During the eclipse period, the front-window temperature drops significantly, causing substantial change in instrument focus. After the end of each eclipse, there is an extended period while the front-window temperature recovers and instrument focus recovers. Throughout the course of the mission, the team has fine-tuned the use of front-window heaters to minimize this recovery time, which currently takes approximately one hour. During this recovery period, periodic focus sweeps are taken to monitor the recovery; focus profiles can be seen in Figure 14 for the Spring 2014 eclipse season.

### Lunar and Planetary Transits

Although they are much less frequent than Earth eclipses, lunar eclipses occur several times per year and cause interruptions in the HMI science data. Although the Moon does not fully occult the solar disk, the HMI ISS must be disabled during these transits, so science-quality data cannot be taken. In addition, the decrease in solar flux decreases the temperature of the front window, which causes a change in focus. The durations of these transits are highly variable, but they typically last between one and three hours.

The planets Mercury and Venus can also pass between the Sun and SDO; this occurred for Mercury in May 2016 and for Venus in June 2012. Transits are useful for calibrating the instrument roll angle, point spread function, and distortion correction (Sections 4.3, 3.3, and 4.2). The HMI ran non-standard observing sequences during all of the transits, which allowed the LoS observables to be produced but not the vector products.

### Instrument Anomalies

Instrument anomalies are caused by occasional and unpredictable problems with the operation of the instrument. Most anomalies result in one or two unusable images, in certain cases, the outages can be hours or days.

#### Corrupt Images

**Figure 15:**
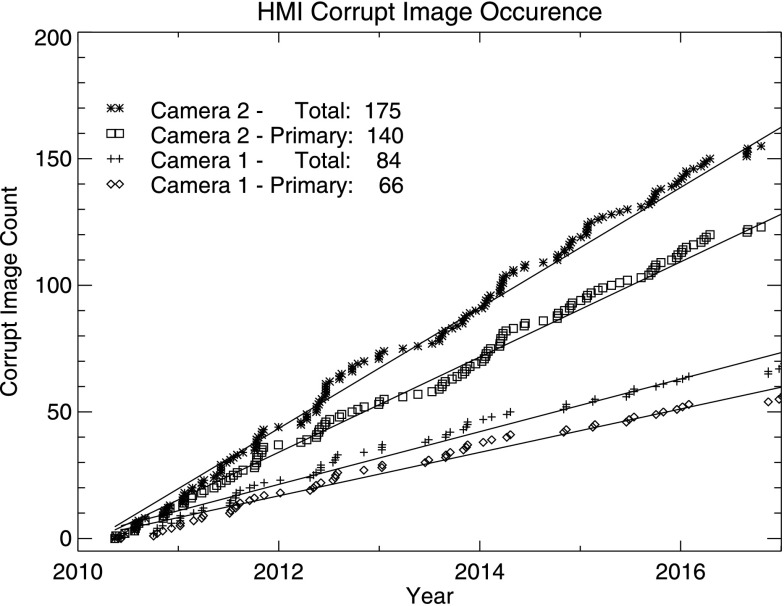
Occurrence of corrupt images as a function of time for the two HMI cameras. The larger total for each camera counts both primary hits and the occasional corruption of the subsequent image.

On occasion, the image file or associated telemetry arrive corrupted at the data-capture system. It is believed that most of these occurrences originate in the camera electronics on the spacecraft, possibly due to cosmic-ray hits. The fraction of images lost this way is roughly one out of every million. The front camera suffers from roughly twice as many instances as the side camera. A cumulative count of corrupt images for each camera is shown in Figure 15. In some instances, corruption of one image affects the data in the following frame, so that the total number of corrupted images is somewhat larger than the number of primary hits.

#### Camera System Errors

Persistent data losses can occur due to errors in the HMI electronics camera interface (CIF) cards or in the data capture–high rate interface (DC-HRI) cards that require intervention from the ground to clear. Errors on the DC-HRI cards involve bit-flips to tables loaded into the field programmable gate arrays (FPGAs) on the cards that determine how the image data are read out. Every table loaded on the cards is checked continuously for parity errors, and alerts are generated when a parity error is detected. The instrument sequencer is then stopped, and the tables are reloaded to clear the parity error. Two types of tables have been affected: the crop tables, which define the area of each image to be stored and downlinked (to save bandwidth, the areas of the image off the solar limb are not downlinked), and the look-up tables that are used for data compression. Errors to the crop tables result in garbled images, while errors to the look-up tables result in one pixel value being changed to another. Garbled images from crop-table errors can be reconstructed, although in some cases, several rows may have missing values. Incorrect data due to look-up table errors generally cannot be fixed, but they do not appreciably affect the quality of the data because only a very few pixels are affected. CIF card errors result in garbled image-header data. A list of the camera anomalies experienced by the HMI is shown in Table 6. When the first anomaly occurred, the error bit set by the parity check was not being monitored, and the effect on the images was simply one partial row of bad values that was difficult to see by eye; consequently, the error was not noticed for almost three weeks. In all subsequent events, the recovery time has been determined by how quickly HMI and SDO personnel can begin commanding the instrument. The majority of camera anomalies have been experienced by Camera 2 (the front/Doppler camera) and they have been increasing somewhat in frequency. Images affected by anomalies are indicated in Level-0 and Level-1 QUALITY bits.

**Table 6:** Camera system and other anomalies experienced by the HMI through December 2017.

Date	Time	Duration	Camera	Event type
22 Dec 2011	08:41 UT	20d 12h 34m	2	Crop table
24 Apr 2013	03:47 UT	6d 9h 46m	–	Processor restart; tuning error
22 Jul 2013	13:21 UT	4h 59m	1	Look-up table
11 Oct 2013	04:54 UT	2h 51m	2	Header error
30 Mar 2014	12:20 UT	4h 27m	1	Look-up table
23 Jun 2014	09:32 UT	2h 45m	1	Header error
23 Mar 2015	23:39 UT	0h 53m	2	Header error
17 May 2015	14:30 UT	8h 20m	–	Processor restart
16 Nov 2015	12:02 UT	2h 39m	2	Header error
16 Feb 2016	19:39 UT	0h 53m	2	Header error
06 Apr 2016	02:04 UT	2h 24m	2	Look-up table
10 Jun 2016	08:26 UT	9h 25m	2	Header error
13 Jun 2016	15:56 UT	1h 47m	2	Look-up table
02 Aug 2016	11:31 UT	1d 13h 23m	–	SDO load shed – intermittent issues until 10 Aug
16 Aug 2016	03:02 UT	13h 20m	2	Header error
16 Nov 2016	20:17 UT	1h 07m	2	Header error
31 Dec 2016	08:49 UT	10h 49m	2	Crop table
11 May 2017	19:45 UT	1h 09m	2	Header error
12 Jun 2017	01:10 UT	14h 41m	2	Look-up table
15 Dec 2017	17:01 UT	1h 27m	2	Header error

#### HMI Reboots and Restarts

The HMI instrument has been rebooted on three separate occasions. The first occurrence was on 24 April 2013, and it was initiated by an error from the processor watchdog that halted the processor. Although most instrument functions were halted, the instrument remained powered on and in the configuration that it was in when the error message was generated. Recovery took 15 hours and 15 minutes. Subsequent analysis of the pre-anomaly telemetry did not reveal what caused the watchdog error message. After the first event, an improperly set sequencer parameter led to errors in the positions of the tunable elements in the Michelsons and Lyot filter, and thus incorrect tuning of the instrument. This error was not corrected until 30 April 2013. A similar event occurred 17 May 2014 with a faster recovery time (8 hours and 20 minutes).

The third HMI reboot involved a full power-down of the instrument when the SDO spacecraft entered Sun-acquisition mode on 2 August 2016 and powered down most of its subsystems, including all three instruments. The HMI instrument was fully powered on and recovered the following day, but science data could not be taken until all of the observatory fine-guidance systems were recovered and calibrated, which did not occur until 4 August 2016.

## Conclusions

The HMI instrument has performed nearly flawlessly since the start of regular SDO operations on 1 May 2010. Nearly 120 million filtergrams have been collected, and more than 98% of all possible 45-second Dopplergrams have been recovered. The HMI instrument and SDO spacecraft have experienced only a very few anomalies, none of which caused extensive data loss.

The HMI team has monitored the instrument continuously to maintain and perfect the calibration of the instrument. Such activities include long-term trending of environmental, optical, spectral, and camera characteristics and analysis of daily, weekly, and quarterly calibration measurements to verify performance.

Trends in slowly varying parameters, such as the instrument focus, filter tuning, and exposure time, are regularly evaluated, and in some cases, operation of the instrument is adjusted to maintain uniform data quality.

For other quantities, such as distortion, wavelength-dependent formation height, and alignment, values are refined and corrections are made to observable quantities as better data become available.

Most of the periodic variations are responses to changes in the thermal environment, largely due to predictable eclipse seasons, planned events, or daily and annual orbital variations. The thermal-control scheme of the instrument was improved to reduce daily and annual variations inside the instrument.

The goal of all of this effort is to provide a complete and uniform-quality record of conditions at the Sun over the solar cycle. The observable quantities – Doppler velocity, intensities, and magnetic field – and downstream higher-level products – convection-zone flow maps, internal rotation, synoptic maps of the photosphere and corona, comprehensive characteristics of active-region evolution – all depend on having a well-calibrated instrument with sufficient information available to eliminate or at least understand the sources of uncertainty in the measurements.

## Figures and Tables

**Figure 16:**
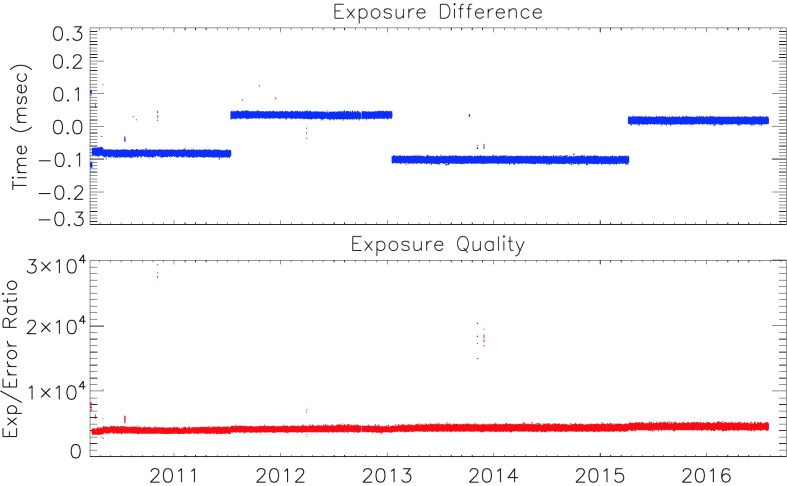
Camera 2 exposure error and quality from 1 May 2010 – 31 July 2016. The *top panel* shows the difference between the commanded and measured exposure times for Camera 2. The *bottom panel* shows the ratio between the exposure time and the standard deviation in the measured exposure times. In each case the values shown are 45-second averages of 12 consecutive exposures that for display purposes are sampled every 12 minutes.

**Figure 17:**
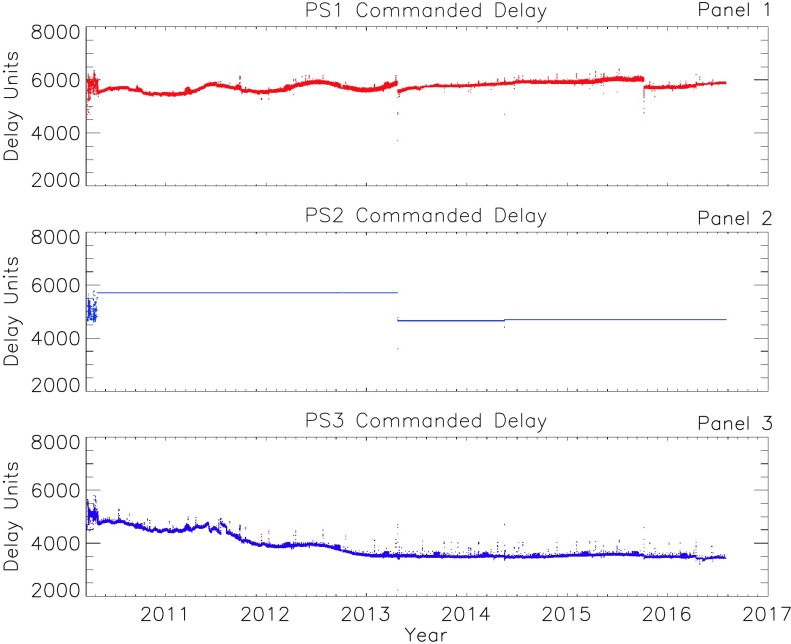
Commanded polarization-selector delay times in engineering delay units. Delay units are approximately microseconds. Values are averaged for 30 minutes.

**Figure 18:**
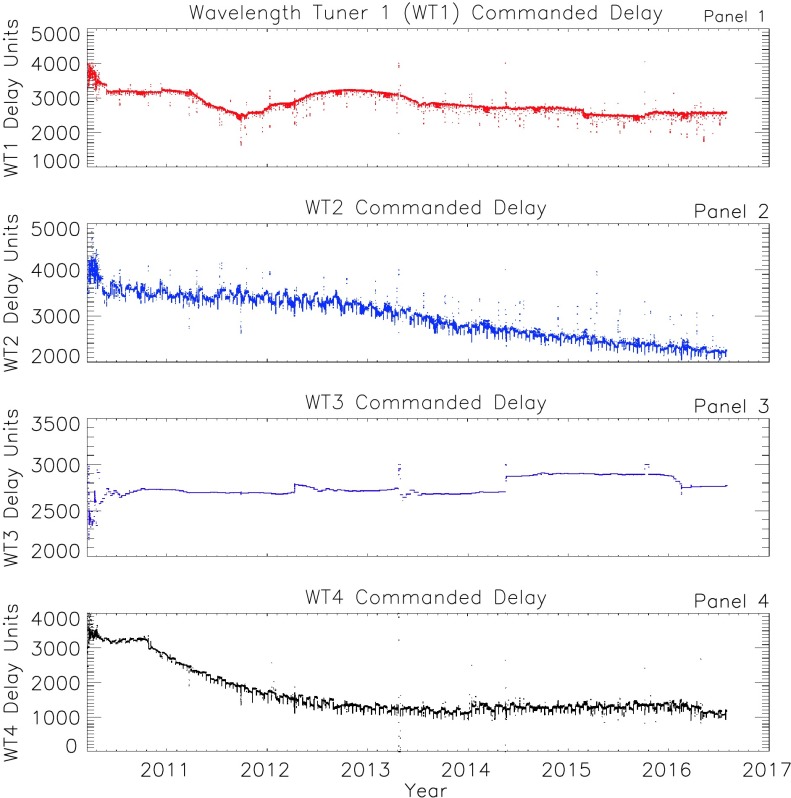
Wavelength tuner delays in engineering delay units.

## Appendix A: Informational Tables Characterizing HMI Performance

The following tables provide more detail than is presented in the main text about Dopplergram recovery rates and common frame lists.

### A.1 HMI Dopplergram Recovery – 72-Day Intervals

Helioseismology requires long uninterrupted time series to determine precise oscillation frequencies. A useful way to characterize the instrument performance is to determine for each 72-day time interval the fraction of good-quality observations recovered by the instrument. The HMI Level-1 mission success requirement for data completeness was to collect at least 95% of all observations during 22 72-day contiguous intervals.

Table 7 shows the Dopplergram recovery rate for the first 37 72-day intervals of the HMI mission, from 30 April 2010 to 14 August 2017. Nominal HMI operations began 1 May 2010, but velocity data were collected early, starting 30 April 2010. For reference, 30 April 2010 is MDI Day 6328. *Perfect* Dopplergrams are those with no QUALITY bits set, *imperfect* Dopplergrams are those with any bit set – most of which are usable for helioseismology. The percentage in the table is the fraction of all possible time slots for which a Dopplergram was recovered. There are 138,240 45-second time slots in each 72-day interval.

**Table 7:** 72-Day HMI Dopplergram Recovery: 30 April 2010 – 18 August 2017

72-day count	GONG months	Start date	End date	# perfect Dopplergrams	# imperfect Dopplergrams	Number missing	Percent recovery
1	153/154	30 Apr 2010	10 Jul 2010	136,034	1736	470	99.66%
2	155/156	11 Jul 2010	20 Sep 2010	133,524	2404	2312	98.33%
3	157/158	21 Sep 2010	01 Dec 2010	129,886	4315	4039	97.08%
4	159/160	02 Dec 2010	11 Feb 2011	135,088	1712	1440	98.96%
5	161/162	12 Feb 2011	24 Apr 2011	130,546	3176	4518	96.73%
6	163/164	25 Apr 2011	05 Jul 2011	136,141	1747	352	99.75%
7	165/166	06 Jul 2011	15 Sep 2011	134,567	2054	1619	98.83%
8	167/168	16 Sep 2011	26 Nov 2011	130,699	3251	4290	96.90%
9	169/170	27 Nov 2011	06 Feb 2012	134,547	2408	1285	99.07%
10	171/172	07 Feb 2012	18 Apr 2012	130,278	4007	3955	97.14%
11	173/174	19 Apr 2012	29 Jun 2012	135,801	2244	195	99.86%
12	175/176	30 Jun 2012	09 Sep 2012	135,210	1754	1276	99.08%
13	177/178	10 Sep 2012	20 Nov 2012	131,729	3065	3446	97.51%
14	179/180	21 Nov 2012	31 Jan 2013	135,983	1306	951	99.31%
15	181/182	01 Feb 2013	13 Apr 2013	131,594	3308	3338	97.59%
16	183/184	14 Apr 2013	24 Jun 2013	123,818	12,581	1841	98.67%
17	185/186	25 Jun 2013	04 Sep 2013	135,469	1473	1298	99.06%
18	187/188	05 Sep 2013	15 Nov 2013	131,065	3331	3844	97.22%
19	189/190	16 Nov 2013	26 Jan 2014	136,018	1308	914	99.34%
20	191/192	27 Jan 2014	08 Apr 2014	131,377	3079	3784	97.26%
21	193/194	09 Apr 2014	19 Jun 2014	135,026	1769	1445	98.95%
22	195/196	20 Jun 2014	30 Aug 2014	135,639	1438	1163	99.16%
23	197/198	31 Aug 2014	10 Nov 2014	131,737	3306	3197	97.69%
24	199/200	11 Nov 2014	21 Jan 2015	135,665	1459	1116	99.19%
25	201/202	22 Jan 2015	03 Apr 2015	130,286	3182	4772	96.55%
26	203/204	04 Apr 2015	14 Jun 2015	135,458	1285	1497	98.92%
27	205/206	15 Jun 2015	25 Aug 2015	135,361	1873	1006	99.27%
28	207/208	26 Aug 2015	05 Nov 2015	131,652	3053	3535	97.44%
29	209/210	06 Nov 2015	16 Jan 2016	133,338	2587	2315	98.33%
30	211/212	17 Jan 2016	28 Mar 2016	131,605	3306	3329	97.59%
31	213/214	29 Mar 2016	08 Jun 2016	134,934	2221	1085	99.22%
32	215/216	09 Jun 2016	19 Aug 2016	128,650	4683	4907	96.45%
33	217/218	20 Aug 2016	30 Oct 2016	130,822	3479	3939	97.15%
34	219/220	31 Oct 2016	10 Jan 2017	136,213	1842	185	99.87%
35	221/222	11 Jan 2017	23 Mar 2017	130,960	3820	3460	97.50%
36	223/224	24 Mar 2017	03 Jun 2017	135,716	1425	1099	99.21%
37	225/226	04 Jun 2017	14 Aug 2017	135,014	1944	1282	99.07%
	Total	30 Apr 2010	14 Aug 2017	4,927,450	102,931	84,499	98.35%

### A.2 Primary HMI Observing and Calibration Frame Lists

The HMI framelist timeline specification (FTS) specifies the sequence, timing, and instrument configuration for exposures in an observation. Each is given a unique identification number: the FTS ID. Table 8 gives information about the primary observing and calibration frame lists used during the mission.

**Table 8:** HMI primary observing and calibration framelists.

FTS ID	Framelist	Duration	Description	When used
Standard observables framelists				
1001	obs_6Cv01	135	Mod C – Standard sequence	1 May – 13 December 2010
1020	obs_6Av02	90	Mod A – Standard sequence	Tested before 1 May 2010
1021	obs_6Cv02	135	Mod C – Standard sequence	13 Dec 2010 – 13 Apr 2016
1022	obs_6Lv02	90	Mod L – Standard sequence	Since 13 April 2016
1026	obs_10v02	150	Mod A – Ten wavelengths	Tested 24 Oct 2014
Daily, weekly, or bi-weekly calibration framelists				
2001	cal_6Cv01	135	Mod-C Darks, continuum, Calmode frames	Daily at 06:00 UT and 18:00 UT, 1 May – 13 Dec 2010
2021	cal_6Cv02	135	Mod-C Darks, continuum, Calmode frames	Daily at 06 and 18 UT, 13 Dec 2010 – 13 Apr 2016
2042	cal_6Lv02	90	Mod-L Darks, continuum, Calmode frames	Daily at 06 and 18 UT, since 13 April 2016
3020	focr_6Cv02	135	Mod-C Reduced focus sweep	Run three times every four weeks, until 13 April 2016
3021	pzt_def_6Cv02	135	Mod-C Obsmode PZT flat	Run twice per week, until 13 April 2016
3022	pzt_cal_6Cv02	135	Mod-C Calmode PZT flat	Run once per week, until 13 April 2016
3023	focus_6Cv02	135	Mod-C Full focus sweep	Run once every four weeks, until 13 April 2016
3027	det_cal_6Cv02	135	Calmode detune sequence	Run once every two weeks (too long for a 90-second framelist)
3040	focr_6Lv02	90	Mod-L Reduced focus sweep	Run three times every four weeks, since 13 April 2016
3041	pzt_def_6Lv02	90	Mod-L Obsmode PZT flat	Run twice per week, since 13 April 2016
3042	pzt_cal_6Lv02	90	Mod-L Calmode PZT flat	Run once per week, since 13 April 2016
3043	focus_6Lv02	90	Mod-L Full focus sweep	Run once every four weeks, since 13 April 2016
Eclipse and calibration-maneuver framelists				
3003	focus_6Cv01	135	Full focus sweep	After Earth eclipses in 2010
3008	focus_6Cv01	135	Full focus sweep, repeating every nine minutes	After Earth eclipses in 2010
3012	focr_6Cv01	135	Reduced focus sweep, repeating every 45 minutes	After Earth eclipses in 2010
3028	focus_6Cv02	135	Full focus sweep, repeating every nine minutes	After Earth eclipses, 2011 – March 2016
3031	focr_6Cv02	135	Reduced focus sweep, repeating every 33 min 45 s	After Earth eclipses, 2011 – March 2016
3128	focus_6Lv02	90	Full focus sweep, repeating every nine minutes	After Earth eclipses, since August 2016
3132	focr_6Lv02	90	Reduced focus sweep, repeating every 45 minutes	After Earth eclipses, since August 2016
4031	focus_off_v02	45	Reduced focus sweep for offpoint maneuvers	During HMI/AIA flat field and EVE FOV maneuvers
4033	rolldopic_v02	45	Set of continuum filtergrams on side camera	During HMI roll maneuvers

The HMI has used two basic observing sequences since commencing regular operations. The Mod-C sequence (FTS IDs 1001 and 1021) was used throughout the prime mission to collect the standard data. On 13 April 2016, after the prime mission ended, HMI switched to a faster sequence, FTS ID 1022, also known as Mod L. The Mod-L sequence requires that images from both cameras be combined to determine the vector-field observables. The part of the frame list for the LoS observables using Camera 2 did not change.

Various calibration sequences are taken on a regular basis to monitor the evolution of the HMI performance. Note that while the JSOC does not keep most older HMI Level-1 data on-line, all of the calibration-related Level-1 data are copied into a data series hmi.lev1_cal, which is permanently on-line.

A more complete list of HMI framelists appears in Tables 9 and 10 of Appendix C.

**Table 9:** HMI framelist timeline specification (FTS) summary – part 1.

FTS ID	FrameList	Duration [seconds]	Repeat	Description
1000	obs_6Av01	90	cont	Obs framelist, Mod A
1001	obs_6Cv01	135	cont	Standard Mod-C framelist until 13 Dec 2010
1002	obs_6Lv01	90	cont	Obs framelist, Mod L
1003	obs_6Mv01	45	cont	Obs framelist, Mod M
1004	obs_6Xv01	45	cont	Obs framelist, Mod X
1020	obs_6Av02	90	cont	Obs framelist, Mod A
1021	obs_6Cv02	135	cont	Std. Mod-C framelist 13 Dec 2010 – 13 Apr 2016
1022	obs_6Lv02	90	cont	Std. Mod-L framelist after 13 Apr 2016
1023	obs_6Mv02	45	cont	Obs framelist, Mod M
1024	obs_6Xv02	45	cont	Obs framelist, Mod X
1025	obs_8Av02	120	cont	Obs framelist, Mod A, 8 wavelengths
1026	obs_10Av02	150	cont	Obs framelist, Mod A, 10 wavelengths
2000	cal_6Cv01	135	24 hr	Daily calibration sequence
2001	cal_6Cv01	135	12 hr	Daily calibration sequence, 1 May – 13 Dec 2010
2002	focr_6Cv01	135	12 hr	Reduced focus sequence
2003	focr_6Cv01	135	24 hr	Reduced focus sequence
2004	focr_6Cv01	135	2.4 hr	Reduced focus sequence
2005	focr_6Cv01	135	1.5 hr	Reduced focus sequence
2020	cal_6Cv02	135	24 hr	Cal sequence (darks, cont. tuned, Calmode frames)
2021	cal_6Cv02	135	12 hr	Daily cal seq. 13 Dec 2010 – 13 Apr 2016
2022	focr_6Cv02	135	12 hr	Reduced focus sequence
2023	focr_6Cv02	135	24 hr	Reduced focus sequence
2024	focr_6Cv02	135	2.4 hr	Reduced focus sequence
2025	focr_6Cv02	135	1.5 hr	Reduced focus sequence
2042	cal_6Lv02	90	12 hr	Daily cal sequence after 13 Apr 2016
2043	cal_6Lv02	90	1 hr	Hourly cal sequence, Mod L
3000	focr_6Cv01	135	cont	Reduced focus sequence
3001	pzt_def_6Cv01	135	cont	PZT flat-field sequence, Obsmode
3002	pzt_cal_6Cv01	135	cont	PZT flat-field sequence, Calmode
3003	focus_6Cv01	135	cont	Full focus sequence
3004	lin_def_6Cv01	135	cont	Linearity test sequence, Obsmode
3005	lin_cal_6Cv01	135	cont	Linearity test sequence, Calmode
3006	det_def_6Cv01	135	cont	Detune sequence, Obsmode
3007	det_cal_6Cv01	135	cont	Detune sequence, Calmode
3020	focr_6Cv02	135	cont	Reduced focus sequence, mod C
3021	pzt_def_6Cv02	135	cont	PZT flat-field sequence, Obsmode
3022	pzt_cal_6Cv02	135	cont	PZT flat-field sequence, Calmode
3023	focus_6Cv02	135	cont	Focus sequence
3024	lin_def_6Cv02	135	cont	Linearity test sequence, Obsmode
3025	lin_cal_6Cv02	135	cont	Linearity test sequence, Calmode
3026	det_def_6Cv02	135	cont	Detune sequence, Obsmode

**Table 10:** HMI FTS summary – part 2.

FTS ID	FrameList	Duration [seconds]	Repeat	Description
3027	det_cal_6Cv02	135	cont	Detune sequence, Calmode
3040	focr_6Lv02	90	cont	Reduced focus sequence, Mod L
3041	pzt_def_6Lv02	90	cont	PZT flat-field sequence, Obsmode, Mod L
3042	pzt_cal_6Lv02	90	cont	PZT flat-field sequence, Calmode, Mod L
3043	focus_6Lv02	90	cont	Full focus sequence, Mod L
3048	cal_6Lv02	90	cont	Mod L calibration sequence, continuous
4000	pzt_def	45	cont	45s cadence PZT flat-field sequence, Obsmode
4001	pzt_cal	45	cont	45s cadence PZT flat-field sequence, Calmode
4002	dop_ic_v01	45	cont	Regular obs seq. on Cam 2; LCP/RCP continuum Cam 1
4003	focus_off_v01	45	cont	Focus sequence for offpoint maneuvers
4004	pl_wob_v01	135	cont	Polarization wobble sequence
4005	wl_wob_v01	180	cont	Wavelength wobble sequence
4006	loop_45	45	cont	Continuous sequence of default filtergrams
4007	loop_90	90	cont	Continuous sequence of default filtergrams
4008	loop_135	135	cont	Continuous sequence of default filtergrams
4009	focus_8_14d	45	cont	Reduced focus sequence
4010	loop_led_45	45	cont	Pre-launch calibration sequence
4011	focus_off_v02	45	cont	Reduced focus sequence for offpoint maneuvers
4012	regulus_135	135	cont	Regulus observing seq.; mix of std. and 3.2s exp.
4013	roll_dop_ic2	45	cont	Roll maneuver seq., continuum filtergrams on Cam 1
4014	cruc_offp_lin	135	cont	Linearity test sequence for EVE cruciform maneuver
4015	regulus_long	135	cont	Regulus observing seq. offpoint with 3.2s exp.
4020	pzt_def	45	cont	45s cadence PZT flat-field sequence, Obsmode
4021	pzt_cal	45	cont	45s cadence PZT flat-field sequence, Calmode
4023	focus_off_v01	45	cont	Reduced focus sequence for offpoint maneuvers
4024	pl_wob_v02	135	cont	Polarization wobble sequence
4025	wl_wob_v02	180	cont	Wavelength wobble sequence
4026	loop_45	45	cont	Continuous sequence of default filtergrams
4027	loop_90	90	cont	Continuous sequence of default filtergrams
4028	loop_135	135	cont	Continuous sequence of default filtergrams
4029	focus_8_14d	45	cont	Reduced focus sequence
4030	loop_led_45	45	cont	Pre-launch calibration sequence
4031	focus_off_v02	45	cont	Focus sequence for offpoint maneuvers
4033	rolldopic_v02	45	cont	Roll man. seq., continuum filtergrams on Cam. 1
4034	venus_2pl_v01	135	cont	Venus transit – std. seq. Cam 2, 2 lin. pol. Cam 1
4035	venus_4pl_v01	135	cont	Venus transit – std. seq. Cam 2, 4 lin. pol. Cam 1
4036	comet_ison	135	cont	Comet ISON seq., 600 ms exposures, default tuning
4037	rolldopic_v03	45	cont	Roll maneuver seq., std. obs Cam 2, continuum Cam 1
4038	rollicscn_v01	45	cont	Roll seq., std. Cam 2, scan cont. to line-core Cam 1
4039	merc_1pl_v01	45	cont	Mercury transit, std. obs Cam 2, 1 lin. pol. Cam 1
4040	cont6Lv01	90	30 min	Std. Mod-L seq. with a set of continuum exp.

## Appendix B: Exposure Time and Filter-Wheel Delays

Figures presented in this section provide more detail about the operation of some components.

### B.3 Exposure Time

As described in Section 4.1, the HMI exposure time is controlled by a mechanical shutter motor that rotates the cut-out sector of an otherwise opaque disk into place with a pause in the *open* position for a commanded time. The difference between the commanded and actual exposure time is monitored at three places in the image plane, and the average over 12 exposures is displayed in the upper panel of Figure 16 for Camera 2. Camera 1 is similar. Typical exposures are 115 – 140 milliseconds. The lower panel shows a measure of the quality of the exposure, which is expressed as the exposure time divided by the standard deviation of the measured exposure times for 12 consecutive exposures. The exposure time can be specified to about 0.12 milliseconds with an observed rms scatter less than 25 microseconds over 45 seconds and a standard deviation of 13.2 microseconds: about a part in 10,000 of the typical exposure. The exposure-time noise is a few times less than the per-pixel photon noise. Individual exposure times are measured with a precision of 1 microseconds and an accuracy better than 4 microseconds; the actual exposure time is reported in the keyword EXPTIME and used in the data analysis pipeline. Shutter noise contributes directly to uncertainty in the observables, because the intensities are used to derive them. The HMI shutter is remarkably uniform and performs much better than the MDI shutter. The few outlier points in the figure occur during non-standard observing sequences.

Figure 17 shows the commanded delay for each of the three polarization selector (PS) wheels. The commanded delays indicate how long it takes for each polarization wheel to move from one selected filter-wheel position to another. Changes on longer timescales indicate changes in resistance or other mechanical issues. Sudden changes are indicative of changes in tuning of the instrument. PS 2 together with wavelength tuning (WT) selector 3 are redundant if one of the wavelength tuners fails. Both are used infrequently.

Figure 18 shows the commanded delay for the four WT filter wheels. WT 1 tunes the narrowband Michelson; WT 2 tunes the wideband Michelson; WT 3 is between the two Michelsons and provides redundancy – it is seldom used; WT 4 tunes the final element of the Lyot filter.

## Appendix C: Framelist and Filtergram IDs and Descriptions

Tables 9 and 10 give information about all of the framelist timeline specifications (FTSs) used by the HMI instrument. Frame lists define a sequence of filtergrams taken for a particular purpose. The duration is indicated in the table. The standard-observables frame list (*e.g.* 1021 or 1022), as well as most others that run only when specifically commanded, repeat continuously unless interrupted. Some calibration sequences (*e.g.* 2021 and 2042) operate on timers that interrupt the regular sequences and execute once at the scheduled repeat interval indicated in the table.

Table 11 identifies the filtergram ID numbers (FIDs) for the various standard instrument tunings. Filtergrams from the standard observing sequence have five-digit FIDs in the range 10000 – 10199. The first (rightmost) digit indicates the polarization setting; the second and third digits give the wavelength tuning. Standard FIDs can be computed as

2 $$ \mathrm{FID} = 10000 + 10 \times \mathrm{WL} + \mathrm{PL} , $$

where WL runs from 0 (the continuum) to 19 (the continuum on the other side of the central wavelength), and PL runs from 0 to 9 as described in the table. Filtergrams used for calibrations usually have four-digit FIDs, which are listed in the table.

**Table 11:** HMI filtergram ID (FID) summary table.

FID	PL index	Polarization state
Standard observing-program FIDs		
10**0	410	Mod A pol 1
10**1	411	Mod A pol 2
10**2	412	Mod A pol 3
10**3	413	Mod A pol 4
10**4	414	*I*+*Q*; linear polarization, 0 deg
10**5	415	*I*−*Q*; linear polarization, 90 deg
10**6	416	*I*+*U*; linear polarization, 45 deg
10**7	417	*I*−*U*; linear polarization, 135 deg
10**8	418	*I*+*V*; left circular polarization
10**9	419	*I*−*V*; right circular polarization

FID	WL index	Wavelength
Standard observing-program FIDs		
1000*	0xff00	$ -344.0~\mbox{m}{\mathring{\mathrm{A}}}$
1001*	0xff01	$ -309.6~\mbox{m}{\mathring{\mathrm{A}}}$
1002*	0xff02	$ -275.2~\mbox{m}{\mathring{\mathrm{A}}}$
1003*	0xff03	$ -240.8~\mbox{m}{\mathring{\mathrm{A}}}$
1004*	0xff04	$ -206.4~\mbox{m}{\mathring{\mathrm{A}}}$
1005*	0xff05	$ -172.0~\mbox{m}{\mathring{\mathrm{A}}}$
1006*	0xff06	$ -137.6~\mbox{m}{\mathring{\mathrm{A}}}$
1007*	0xff07	$ -103.2~\mbox{m}{\mathring{\mathrm{A}}}$
1008*	0xff08	$ -68.9~\mbox{m}{\mathring{\mathrm{A}}}$
1009*	0xff09	$ -34.4~\mbox{m}{\mathring{\mathrm{A}}}$
1010*	0xff0a	$ 0.0~\mbox{m}{\mathring{\mathrm{A}}}$
1011*	0xff0b	$ 34.4~\mbox{m}{\mathring{\mathrm{A}}}$
1012*	0xff0c	$ 68.8~\mbox{m}{\mathring{\mathrm{A}}}$
1013*	0xff0d	$ 103.2~\mbox{m}{\mathring{\mathrm{A}}}$
1014*	0xff0e	$ 137.6~\mbox{m}{\mathring{\mathrm{A}}}$
1015*	0xff0f	$ 172.0~\mbox{m}{\mathring{\mathrm{A}}}$
1016*	0xff10	$ 206.4~\mbox{m}{\mathring{\mathrm{A}}}$
1017*	0xff11	$ 240.8~\mbox{m}{\mathring{\mathrm{A}}}$
1018*	0xff12	$ 275.2~\mbox{m}{\mathring{\mathrm{A}}}$
1019*	0xff13	$ 309.6~\mbox{m}{\mathring{\mathrm{A}}}$

FID	Description
Calibration and special-observation FIDs	
5000	Regular dark frame
5001	Throwaway dark frame
5002	General image
5003	Linearity test
5004	Linearity test with darks
5101 – 5116	Focus-sequence filtergrams
5117	Calmode filtergram
Wavelength-calibration FIDs	
6000 – 6026	Standard detune (100 – 126)
6027 – 6030	Extra positions for 31-frame detune
6101 – 6196	Wobble sequence (WLID = 1 – 96)
620 – -6296	Wobble + 20 sequence
Polarization-calibration FIDs	
7101 – 7172	Wobble sequence (WLID = 1 – 72)
7201 – 7272	Wobble sequence (WLID = 175 – 246)

## Appendix D: Level-0 FITS Keywords

Table 12 lists the Level-0 keywords associated with details of the image.

**Table 12:** HMI Level-0 keywords – image details.

Keyword	Type	Description
BLD_VERS	string	Build version: from jsoc_version.h
ORIGIN	string	Constant: location where file is made – SDO/JSOC-SDP
DATE	time	Date and time of processing; ISO 8601
TELESCOP	string	Constant: for HMI: SDO/HMI
INSTRUME	string	HMI light path: HMI_SIDE1 or HMI_FRONT2
DATE-OBS	time	Date when observation started; ISO 8601
T_OBS	time	Observation time
CAMERA	integer	HMI camera numeric identifier: 1 or 2
IMG_TYPE	string	Image type: LIGHT or DARK
EXPTIME	double	Exposure duration: shutter open time in seconds
EXPSDEV	float	Exposure standard deviation in seconds
WAVELNTH	integer	Constant: for HMI = 6173.3 angstrom
WAVEUNIT	string	Constant: Wavelength unit = angstrom
FSN	integer	FSN – Filtergram Sequence Number
FID	integer	FID – Filtergram ID
TLMDSNAM	string	Telemetry data series based on data packet time
IMGFPT	time	Time stamp of the first image data packet
IMGAPID	integer	Application ID of the science data packets
TAPCODE	integer	Take A Picture code for the camera readout
BITSELID	integer	Bit select ID; r-value for the data compression
COMPID	integer	Compression ID; data compression n- and k-values
CROPID	integer	Crop table ID used in data downlink
LUTID	integer	Look-up table ID used in data downlink
NPACKETS	integer	Number of packets in image
NERRORS	integer	Number of decompression errors in image
EOIERROR	short	End Of Image error; last pixel error occurred in image
HEADRERR	short	Header error occurred in image
OVERFLOW	short	Data overflow error occurred in image
QUALITY	integer	Quality keyword
TOTVALS	integer	Expected number of data values [pixels] in image
DATAVALS	integer	Actual number of data values in image
MISSVALS	integer	Missing values: TOTVALS – DATAVALS
PERCENTD	float	Percent data; 100×DATAVALS / TOTVALS
DATAMIN	short	Minimum value of all pixels
DATAMAX	short	Maximum value of all pixels
DATAMEDN	short	Median value of all pixels
DATAMEAN	float	Mean value of all pixels
DATARMS	float	Rms deviation from the mean value of all pixels
DATASKEW	float	Skewness from the mean value of all pixels
DATAKURT	float	Kurtosis of all pixels

Table 13 describes the Level-0 keywords associated with the status of the onboard image status packet (ISP) sequencer.

**Table 13:** HMI Level-0 keywords – image status packet (ISP) sequencer status.

Keyword	Type	Description
ISPSNAME	string	Image Status Packet (ISP) series name
ISPPKTIM	time	Prime key value for the ISP record
ISPPKTVN	string	ISP packet version
HSQFGSN	integer	Unique serial number for each image (filtergram) taken
HSQFGID	integer	Filtergram identifier parameters
HCAMID	integer	Current light-path identifier
HSHIEXP	integer	Current shutter-exposure value in milliseconds
HOBITSEC	integer	TAI seconds of the shutter-move start time
HOBITSS	integer	Subseconds field of the shutter-move start time
HWLTNSET	string	Image Stabilization System (ISS) loop status
HSQSTATE	string	Sequencer state: IDLE, SELECTING, or PROCESSING
HSEQERR	string	Sequence error message of the last sequencer error
HFLREFTM	integer	Current framelist reference start time
HFLRELTM	integer	Current frame time: milliseconds from reference time
HFLID	integer	Framelist identification number
HOBLSTID	integer	Observation list identification number
HFLPSITN	integer	Position number of the current frame in framelist
HSQFCNT	integer	Number of frames taken after restarting the sequence
HFLLNGTH	short	Total number of frames in the current framelist (FTS)
HFLRPTCT	integer	Cadence periods to repeat for the current FTS
HFLRPTNM	integer	Repeat number of the active framelist
HFLSKPCT	integer	Cadence periods to skip for the current FTS
HFTSACID	integer	Identification number of the current active FTS
HFTSCDMK	integer	Number of cadence periods after restarting the sequence
HFTSINFO	integer	FTS status information
HSQEIDX	integer	Current exposure index number
HIMGCFID	integer	Current image configuration identification number
HCFTID	integer	Current focus position identification number
HPLTID	integer	Current polarization selector identification number
HWLTID	integer	Current the wavelength tuning identification number
HWLSTIDX	integer	Current wavelength set index number
HGP1RGST	integer	General purpose register 1 (set by command)
HGP2RGST	integer	General purpose register 2 (set by command)

Table 14 describes the Level-0 keywords associated with the status of the onboard mechanisms.

**Table 14:** HMI Level-0 keywords – ISP mechanism parameters.

Keyword	Type	Description
HSHMIOPB	float	Shutter timer open value for bottom position
HSHMIOPM	float	Shutter timer open value for middle position
HSHMIOPT	float	Shutter timer open value for top position
HSHMICLB	float	Shutter close timer value for bottom position
HSHMICLM	float	Shutter close timer value for middle position
HSHMICLT	float	Shutter close timer value for top position
HCF1ENCD	integer	Encoder value returned from CF1 mechanism
HCF2ENCD	integer	Encoder value returned from CF2 mechanism
HPS1ENCD	integer	Encoder value returned from PS1 mechanism
HPS2ENCD	integer	Encoder value returned from PS2 mechanism
HPS3ENCD	integer	Encoder value returned from PS3 mechanism
HWT1ENCD	integer	Encoder value returned from WT1 mechanism
HWT2ENCD	integer	Encoder value returned from WT2 mechanism
HWT3ENCD	integer	Encoder value returned from WT3 mechanism
HWT4ENCD	integer	Encoder value returned from WT4 mechanism
HCF1POS	integer	Commanded target position for CF1 mechanism
HCF2POS	integer	Commanded target position for CF2 mechanism
HPL1POS	integer	Commanded target position for PS1 mechanism
HPL2POS	integer	Commanded target position for PS2 mechanism
HPL3POS	integer	Commanded target position for PS3 mechanism
HWL1POS	integer	Commanded target position for WT1 mechanism
HWL2POS	integer	Commanded target position for WT2 mechanism
HWL3POS	integer	Commanded target position for WT3 mechanism
HWL4POS	integer	Commanded target position for WT4 mechanism

## Appendix E: Level-0 QUALITY-Keyword Summary

The HMI uses a QUALITY keyword to describe properties of the data and data processing at each level of reduction. Generally, a bit in the keyword is set only when there is a problem.

Table 15 gives the meaning of the bits put into the QUALLEV0 keyword for each filtergram record in the Level-0 data series determined by the ingest_lev0 processing module. Bit 0 is the low bit (0x01)

**Table 15:** HMI Level-0 quality summary.

Quality	Bit Mask	Description
Q_OVFL	0x00000001	Overflow flag set
Q_HDRERR	0x00000002	Header error flag set
Q_CMPERR	0x00000004	Compression error in image
Q_LPXERR	0x00000008	Last pixel error
Q_NOISP	0x00000010	No ISP; FSN ≠ HSQFGSN
Q_MISSI	0x00000020	Missing image
Q_CORRUPT	0x00000040	Corrupt image; FSN = 469769216 or 0x1c001c00
Q_INVALTIME	0x00000080	HOBITSEC = 0; T_OBS = 1958.01.01_00:00:00_UTC
Q_MISS0	0x00000100	MISSVALS > 0
Q_MISS1	0x00000200	MISSVALS > 0.01*TOTVALS
Q_MISS2	0x00000400	MISSVALS > 0.05*TOTVALS
Q_MISS3	0x00000800	MISSVALS > 0.25*TOTVALS
	0x00001000	Unused
	0x00002000	Unused
	0x00004000	Unused
Q_CAM_ANOM	0x00008000	Camera anomaly; entered manually
Q_DARK	0x00010000	Dark image
Q_ISSOPEN	0x00020000	ISS loop open; HWLTNSET = 'OPEN'
Q_HCF1ENCD	0x00040000	Focus/Cal Motor 1 Error
		HCF1ENCD ≠ HCF1POS ± 1
Q_HCF2ENCD	0x00080000	Focus/Cal Motor 2 Error
		HCF2ENCD ≠ HCF2POS ± 1
Q_HPS1ENCD	0x00100000	Polarization Motor 1 Error
		HPS1ENCD ≠ HPL1POS ± 1
Q_HPS2ENCD	0x00200000	Polarization Motor 2 Error
		HPS2ENCD ≠ HPL2POS ± 1
Q_HPS3ENCD	0x00400000	Polarization Motor 3 Error
		HPS3ENCD ≠ HPL3POS ± 1
Q_HWT1ENCD	0x00800000	Wavelength Motor 1 Error
		HWT1ENCD ≠ HWL1POS ± 1
Q_HWT2ENCD	0x01000000	Wavelength Motor 2 Error
		HWT2ENCD ≠ HWL2POS ± 1
Q_HWT3ENCD	0x02000000	Wavelength Motor 3 Error
		HWT3ENCD ≠ HWL3POS ± 1
Q_HWT4ENCD	0x04000000	Wavelength Motor 4 Error
		HWT4ENCD ≠ HWL4POS ± 1
	0x08000000	Unused
Q_GPREGBIT0	0x10000000	HGP1RGST bit 0 set
Q_GPREGBIT1	0x20000000	HGP1RGST bit 1 set
Q_REOPENED	0x40000000	Image reopened during reconstruction
		NPACKETS value may be incorrect
Q_MISSALL	0x80000000	Data are completely missing
		High bit

## Appendix F: Level-1 FITS Keywords

Table 16 describes Level-1 keywords associated with WCS coordinates and SDO orbit parameters.

**Table 16:** HMI Level 1 keywords – WCS and orbit parameters.

Keyword	Type	Description
T_OBS_step	double	T_OBS step (constant); 1.000000 second
T_OBS_epoch	time	T_OBS epoch (constant); 1977.01.01_00:00:00_TAI
OSCNMEAN	float	Mean value of removed overscan rows
OSCNRMS	float	Rms deviation from the mean value of overscan rows
FLAT_REC	string	Flat field series record pointer
NBADPERM	integer	Count of permanent bad pixels
NBADTOT	integer	Count of total bad pixels
CTYPE1	string	Typically HPLN-TAN (SOLARX)
CUNIT1	string	Typically arcseconds
CRVAL1	float	Image scale in the x-direction; arcseconds pixel^−1^
CDELT1	float	Image scale in the x-direction; arcseconds pixel^−1^
CRPIX1	float	Location of Sun center in CCD x direction; pixel
CTYPE2	string	Typically HPLT-TAN (SOLARY)
CUNIT2	string	Typically arcseconds
CRVAL2	float	Image scale in the x-direction; arcseconds pixel^−1^
CDELT2	float	Image scale in the y-direction; arcseconds pixel^−1^
CRPIX2	float	Location of Sun center in CCD y-direction; pixel
CROTA2	float	INST_ROT + SAT_ROT; degrees
R_SUN	float	Radius of the Sun on the CCD detector; pixels
MPO_REC	string	Master Pointing series record pointer
INST_ROT	float	Master pointing CCD rotation wrt SDO Z axis; degrees
IMSCL_MP	float	Master pointing image scale; arcseconds pixels^−1^
X0_MP	float	Master pointing X0 sun center in CCD frame; pixels
Y0_MP	float	Master pointing Y0 sun center in CCD frame; pixels
RSUN_LF	float	Limb fit solar radius; pixels
X0_LF	float	Limb fit X0 Sun center in CCD frame; pixels
Y0_LF	float	Limb fit Y0 Sun center in CCD frame; pixels
CALVER32	integer	Height of formation correction version
ASD_REC	string	Ancillary Science Data series record pointer
SAT_Y0	float	Position of solar center wrt the SDO −Y-axis; arcseconds
SAT_Z0	float	Position of solar center wrt the SDO +Z-axis; arcseconds
SAT_ROT	float	Angle of solar pole wrt the SDO +X-axis; degrees
ACS_MODE	string	ACS pointing mode; ACS_AN_ACS_MODE
ACS_ECLP	string	ACS eclipse flag; ACS_AN_FLAG_CSS_ECLIPSE
ACS_SUNP	string	ACS Sun presence flag; ACS_AN_FLAG_DSS_SUNPRES
ACS_SAFE	string	ACS safe hold flag; ACS_AN_FLAG_ACE_INSAFEHOLD
ACS_CGT	string	ACS Controlling Guide Telescope ID; ACS_AN_NUM_CGT
ORB_REC	string	Orbit vector series record pointer
DSUN_REF	double	Reference distance to Sun (constant): 149,597,870,691.0 m
DSUN_OBS	double	Distance from SDO to Sun center; m
RSUN_REF	double	Reference radius of the Sun (constant): 696,000,000.0 m
RSUN_OBS	double	Apparent radius of the Sun seen by SDO; arcseconds
GAEX_OBS	double	Geocentric Inertial X-position; m
GAEY_OBS	double	Geocentric Inertial Y-position; m
GAEZ_OBS	double	Geocentric Inertial Z-position; m
HAEX_OBS	double	Heliocentric Inertial X-position; m
HAEY_OBS	double	Heliocentric Inertial Y-position; m
HAEZ_OBS	double	Heliocentric Inertial Z-position; m
OBS_VR	double	Speed of observer in radial direction; m s^−1^
OBS_VW	double	Speed of observer in solar-west direction; m s^−1^
OBS_VN	double	Speed of observer in solar-north direction; m s^−1^
CRLN_OBS	float	Carrington longitude of the observer; degrees
CRLT_OBS	float	Carrington latitude of the observer; degrees
CAR_ROT	integer	Carrington rotation number of CRLN_OBS
HGLN_OBS	float	Stonyhurst heliographic longitude of the observer; degrees
HGLT_OBS	float	Stonyhurst heliographic latitude of the observer; degrees

## Appendix G: Level-1 QUALITY-Keyword Summary

Table 17 provides the meaning of the bits put into the Level-1 QUALITY keyword for each filtergram in the Level-1 data series by the build_lev1 processing module. Bit 0 is the low bit (0x01)

**Table 17:** HMI Level-1 quality summary.

Quality	Bit Mask	Description
Q_NOFLAT	0x00000001	Flat field not available or error
Q_NOORB	0x00000002	Orbit data not available or error
Q_NOASD	0x00000004	Ancillary science data not available or error
Q_NOMPD	0x00000008	Master pointing data not available or error
Q_NOLIMB	0x00000010	Limb fit error
	0x00000020	Unused
	0x00000040	Unused
Q_CAM_ANOM1	0x00000080	Camera anomaly
Q_1_MISS0	0x00000100	MISSVALS > 0
Q_1_MISS1	0x00000200	MISSVALS > 0.01×TOTVALS
Q_1_MISS2	0x00000400	MISSVALS > 0.05×TOTVALS
Q_1_MISS3	0x00000800	MISSVALS > 0.25×TOTVALS
Q_NOACS_SCI	0x00001000	ACS_MODE ≠ 'SCIENCE'
Q_ACS_ECLP	0x00002000	ACS_ECLP = 'YES'; spacecraft eclipse flag
Q_ACS_SUNP	0x00004000	ACS_SUNP = 'NO'; no Sun presence
Q_ACS_SAFE	0x00008000	ACS_SAFE = 'YES'; safemode flag set
Q_IMG_TYPE	0x00010000	Dark image
Q_LOOP_OPEN	0x00020000	ISS Loop Open
Q_CAL_IMG	0x00040000	Calibration image
Q_CALM_IMG	0x00080000	HMI calibration mode image
Q_AIA_FOOR	0x00100000	Not used for HMI
Q_AIA_REGF	0x00200000	Not used for HMI
Q_THERM_RECOV	0x00400000	HMI thermal recovery
Q_LUNAR_TRAN	0x00800000	HMI lunar transit
	0x01000000	Unused
	0x02000000	Unused
	0x04000000	Unused
	0x08000000	Unused
	0x10000000	Unused
	0x20000000	Unused
Q_NRT	0x40000000	Near Real Time mode
Q_MISSALL	0x80000000	Image not available; high bit

## Appendix H: HMI Observables QUALITY-Keyword Summary

The keyword QUALITY is provided for each HMI data product at each processing level. Observables are constructed from many filtergrams, so QUALITY may depend on information from any of the contributing images.

Table 18 describes QUALITY-bits for the 45-second LoS observables that indicate why a data record is missing. The value of QUALITY will be negative (top bit set) if data are missing for one of the reasons specified in certain other bits. LoS observables include 45-second Dopplergrams, LoS magnetic field, line depth, and continuum intensity. Filtergrams at multiple wavelengths and polarization states from three consecutive 45-second intervals contribute to each data record. The 45-second observables depend on filtergrams only from Camera 2.

**Table 18:** HMI line-of-sight observable processing-failure quality-bit summary.

QUALITY-bit name	Bit mask	Description
QUAL_NODATA	0x80000000	No LoS observables image was produced (empty record created, with no data segment. Most keywords have default value)
QUAL_TARGETFILTERGRAMMISSING	0x40000000	No filtergram found near target time
QUAL_NOINTERPOLATEDKEYWORDS	0x20000000	Could not interpolate required keywords at target time
QUAL_NOFRAMELISTINFO	0x10000000	Could not identify observables framelist used
QUAL_WRONGCADENCE	0x08000000	Framelist cadence required time does not match the expected value
QUAL_WRONGTARGET	0x04000000	Target filtergram does not belong to the current framelist
QUAL_MISSINGLEV1D	0x02000000	Not enough lev1d filtergrams to produce observable
QUAL_MISSINGKEYWORDLEV1D	0x01000000	Could not read some required keywords in lev1d data
QUAL_WRONGWAVELENGTHNUM	0x00800000	Number of wavelengths in the lev1d records is incorrect
QUAL_MISSINGKEYWORDLEV1P	0x00400000	Could not read some required keywords in the lev1p data
QUAL_NOLOOKUPRECORD	0x00200000	Could not find a record for look-up tables for the MDI-like algorithm
QUAL_NOLOOKUPKEYWORD	0x00100000	Could not read keywords of the look-up tables for the MDI-like algorithm
QUAL_NOTENOUGHINTERPOLANTS	0x00080000	Not enough interpolation points for the temporal interpolation at a given wavelength and polarization
QUAL_INTERPOLATIONFAILED	0x00040000	Temporal interpolation failed (no lev1d record was produced)
QUAL_MISSINGLEV1P	0x00020000	Not enough lev1p records to produce an observable
QUAL_NOCOEFFKEYWORD	0x00000200	Could not read keywords of the polynomial coefficient series for the correction of the MDI-like algorithm
QUAL_NOCOEFFPRECORD	0x00000080	Could not find a record for the polynomial coefficient for the correction of the MDI-like algorithm, or could not access the keywords of a specific record

Data records with no known issues have QUALITY = 0. Bit 0 is the low bit (0x01).

Table 19 provides similar information for the bits in the vector-observable QUALITY keyword. The primary observable is the Stokes data series hmi.S_720s. The temporal interpolation is much longer than for the 45-second data products, including data collected over a nearly 20-minute time span from one or both HMI cameras. The other HMI 720-second observables are computed from the Stokes observable, so share the same keyword.

**Table 19:** HMI stokes *I Q U V* observable processing-failure QUALITY-bit summary (any cadence).

QUALITY-bit name	Bit mask	Description
QUAL_NODATA	0x80000000	Not all *I Q U V* filtergrams produced (some or all data segments missing)
QUAL_TARGETFILTERGRAMMISSING	0x40000000	No target filtergram found near target time
QUAL_NOINTERPOLATEDKEYWORDS	0x20000000	Could not interpolate some required keywords at target time
QUAL_NOFRAMELISTINFO	0x10000000	Could not recognize observables framelist
QUAL_WRONGCADENCE	0x08000000	Cadence corresponding to the framelist does not match the expected value provided by user
QUAL_WRONGFRAMELISTSIZE	0x04000000	Current framelist size does not match value from the command line
QUAL_WRONGNPOL	0x02000000	Current framelist npol does not match value from the command line
QUAL_WRONGPOLTYPE	0x01000000	Current framelist does not allow for the production of *I Q U V*
QUAL_WRONGTARGET	0x00800000	Target filtergram does not belong to the current framelist
QUAL_ERRORFRAMELIST	0x00400000	Filtergrams not where they should be in the framelist
QUAL_WRONGWAVELENGTHNUM	0x00200000	Number of wavelengths in the lev1d records is not correct
QUAL_NOLOOKUPRECORD	0x00100000	Could not find record for look-up tables for MDI-like algorithm (currently unused)
QUAL_NOLOOKUPKEYWORD	0x00080000	Could not read keywords of look-up tables for MDI-like algorithm (currently unused)
QUAL_NOTENOUGHINTERPOLANTS	0x00040000	Not enough points for the temporal interpolation at a given wavelength and polarization
QUAL_INTERPOLATIONFAILED	0x00020000	temporal interpolation routine failed

Table 20 provides information for the additional bits in the vector-observable QUALITY keyword for data that may be acceptable in certain circumstances, but may be of lesser quality due to inclusion of fewer filtergrams or noisier data.

**Table 20:** HMI stokes *I Q U V*-observable poor-QUALITY bits.

QUALITY-bit Name	Bit Mask	Description
QUAL_LOWINTERPNUM	0x00010000	Too few averaging points or two interpolation points separated by more than the cadence
QUAL_LOWKEYWORDNUM	0x00008000	Some keywords (*e.g.* CROTA2, DSUN_OBS, and CRLT_OBS) could not be interpolated properly, closest-neighbor approximation used
QUAL_ISSTARGET	0x00004000	ISS Loop open for one or several filtergrams used to produce observable
QUAL_NOTEMP	0x00002000	Cannot read temperatures needed for polarization calibration (default temperature used)
QUAL_NOGAPFILL	0x00001000	Code could not properly gap-fill all Lev-1 filtergrams
QUAL_LIMBFITISSUE	0x00000800	Some Lev-1 records discarded because R_SUN, CRPIX1, or CRPIX2 were missing or too different from median
QUAL_NOCOSMICRAY	0x00000400	Some cosmic-ray hit lists could not be read for level 1 filtergrams
QUAL_ECLIPSE	0x00000200	At least one lev1 record taken during an eclipse
QUAL_LARGEFTSID	0x00000100	HFTSACID of target filtergram >4000, which adds noise to observable
QUAL_TEMPERROR	0x00000080	Code error discovered that will be corrected in later processing versions, see notes at jsoc2.stanford.edu/doc/data/hmi/Quality_Bits
QUAL_POORQUALITY	0x00000020	WARNING poor quality: be careful when using due to eclipse, transit, thermal recovery, open ISS, or other

## Footnotes

1 See jsoc.stanford.edu/doc/data/hmi/hmi_retuning.txt.
